# Silver Nanoparticles Impact Biofilm Communities and Mussel Settlement

**DOI:** 10.1038/srep37406

**Published:** 2016-11-21

**Authors:** Jin-Long Yang, Yi-Feng Li, Xiao Liang, Xing-Pan Guo, De-Wen Ding, Demin Zhang, Shuxue Zhou, Wei-Yang Bao, Nikoleta Bellou, Sergey Dobretsov

**Affiliations:** 1 Key Laboratory of Exploration and Utilization of Aquatic Genetic Resources, Shanghai Ocean University, Ministry of Education, China; 2 Marine Ecology Research Center, The First Institute of Oceanography, State Oceanic Administration, Qingdao, China; 3 Collaborative Innovation Center for Zhejiang Marine High-efficiency and Healthy Aquaculture, Ningbo, China; 4 Department of Materials Science, State Key Laboratory of Molecular Engineering of Polymers, Advanced Coatings Research Center of Ministry of Education of China, Fudan University, Shanghai, China; 5 Institute of Marine Science and Technology, Yangzhou University, Yangzhou, China; 6 Hellenic Centre for Marine Research, Institute of Oceanography, Athens, Greece; 7 Department of Marine Science and Fisheries, College of Agricultural and Marine Sciences, Sultan Qaboos University, Muscat, Oman; 8 Center of Excellence in Marine Biotechnology, Sultan Qaboos University, Muscat, Oman

## Abstract

Silver nanoparticles (AgNPs) demonstrating good antimicrobial activity are widely used in many fields. However, the impact of AgNPs on the community structures of marine biofilms that drive biogeochemical cycling processes and the recruitment of marine invertebrate larvae remains unknown. Here, we employed MiSeq sequencing technology to evaluate the bacterial communities of 28-day-old marine biofilms formed on glass, polydimethylsiloxane (PDMS), and PDMS filled with AgNPs and subsequently tested the influence of these marine biofilms on plantigrade settlement by the mussel *Mytilus coruscus*. AgNP-filled PDMS significantly reduced the dry weight and bacterial density of biofilms compared with the glass and PDMS controls. AgNP incorporation impacted bacterial communities by reducing the relative abundance of Flavobacteriaceae (phylum: Bacteroidetes) and increasing the relative abundance of Vibrionaceae (phylum: Proteobacteria) in 28-day-old biofilms compared to PDMS. The settlement rate of *M. coruscus* on 28-day-old biofilms developed on AgNPs was lower by >30% compared to settlement on control biofilms. Thus, the incorporation of AgNPs influences biofilm bacterial communities in the marine environment and subsequently inhibits mussel settlement.

Recently, the application of nanomaterials such as copper, titanium and silver has expanded considerably in various fields^1^. Due to their efficacious antimicrobial actions against bacteria, diatoms and viruses^1^,^2^,^3^,^4^, silver nanoparticles (AgNPs) have been applied in the food sector^5^, medical devices^6^, textile finishings^7^ and water treatment systems^8^. While the impact of nanoparticles (NPs) on aquatic microbes has been investigated^9^, few studies^10^,^11^,^12^ have been conducted in the marine environment to examine the impact of AgNPs on natural biofilms that play key roles in biogeochemical cycling processes and larval and spore recruitment^13^,^14^.

In the marine environment, biofilms are ubiquitous on submerged substrata^15^. Marine biofilms comprise many species of bacteria and unicellular eukaryotes (e.g., diatoms and flagellates)^15^ but are dominated by multiple bacterial and diatom species^15^,^16^. Marine biofilms are crucial for larval recruitment for some invertebrates^14^,^15^,^16^,^17^,^18^, and the bacteria in biofilms are viewed as sources of settlement-inducing cues for invertebrate larvae^18^,^19^.

Bacterial strains isolated from biofilms have been shown to induce the larval recruitment of invertebrates, such as the coral *Pocillopora damicornis*^20^, the tubeworm *Hydroides elegans*^21^, and the mussel *Mytilus galloprovincialis*^22^. However, the presence of such bacterial strains in natural biofilms does not always explain the recruitment activity of biofilms^23^,^24^. Previous studies have demonstrated the importance of complex microbial communities during invertebrate larval settlement, such as that of *H. elegans*^23^,^25^,^26^, *Balanus amphitrite*^24^,^26^,^27^, *B. trigonus*^28^, and *Rhopaloeides odorabile*^28^. In the case of mussels, variances in biofilm composition result in differential larval settlement^29^. Therefore, it is important to investigate changes in marine biofilm communities and subsequent effects on mussel settlement to understand the possible environmental impact of NPs.

The mussel *Mytilus coruscus* is an important aquaculture and fouling bivalve in China^30^,^31^,^32^. In this study, MiSeq sequencing technology was employed to ascertain the influence of AgNPs on the formation of marine biofilms on glass and polydimethylsiloxane (PDMS) substrata in a marine environment as well as the subsequent influence of these bacterial communities on *M. coruscus* settlement. Glass is widely used to investigate biofilm formation in marine environments^20^,^21^,^29^,^33^, and PDMS is a known fouling release material employed in antifouling studies with marine invertebrates^34^,^35^,^36^. The purpose of this study was to determine whether biofilm communities differ on substrata with and without AgNPs and if variations in bacterial communities alter settlement-inducing activity.

## Results

### Settlement bioassay

**Figure 1:**
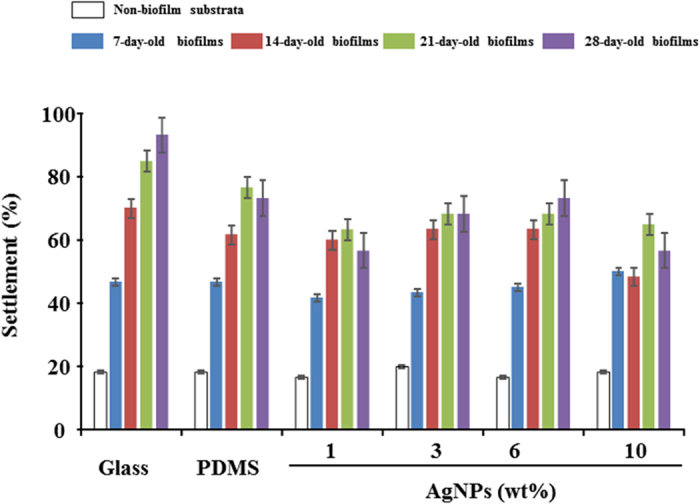
Percentage of *M. coruscus* plantigrades on non-biofilm and biofilm substrata. Non-biofilm substrata included glass, PDMS, and PDMS filled with AgNPs. Data are the mean ± standard error (SE) (n = 6).

No differences were observed in the settlement of *M. coruscus* plantigrades on surfaces lacking biofilms (Fig. 1, *p* = 0.98). Similar tendencies were also observed for 7-day-old biofilms. For 14-day-old biofilms, the plantigrade settlement rate on AgNP-filled PDMS was similar to the settlement rates on glass and PDMS (controls), with the exception of PDMS filled with 10 weight% (wt%) AgNPs (*p* < 0.0001). After 21 days, the biofilms that developed on PDMS filled with 10 wt% AgNPs induced significantly lower settlement than those on glass (*p* < 0.0001). After 28 days, the settlement rates were significantly lower for biofilms on PDMS filled with 1 wt% and 10 wt% AgNPs compared with those on glass and PDMS (*p* < 0.0001).

### Biomass of marine biofilms

**Figure 2:**
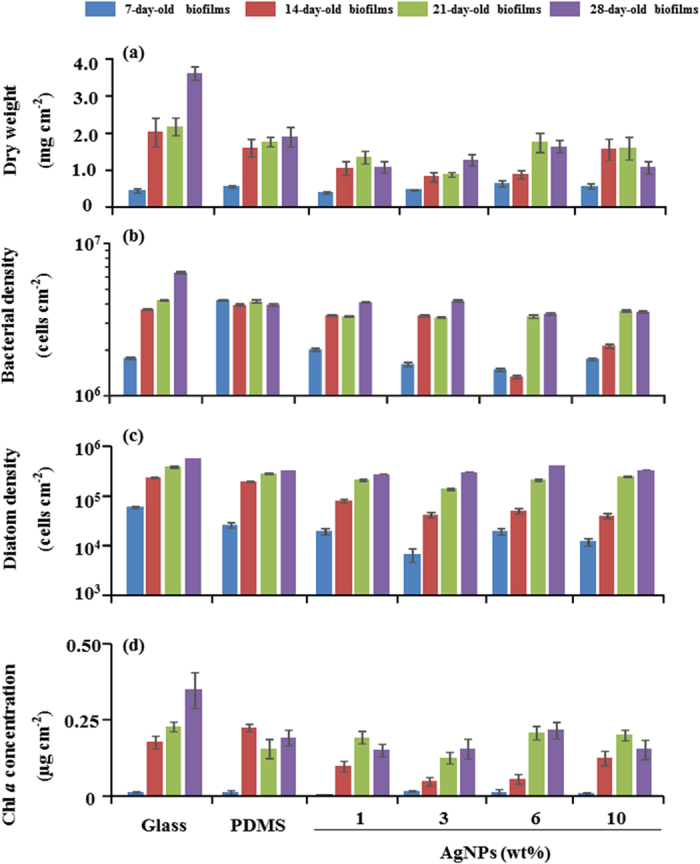
Dry weights (**a**), bacterial densities (**b**), diatom densities (**c**) and chl *a* concentrations (**d**) of natural biofilms on different substrata. Data are the mean ± SE (n = 6).

There were no differences between the dry weights of 7-day-old biofilms on differentially treated surfaces (Fig. 2a). The dry weights of biofilms that developed after 14 d on PDMS filled with 3 wt% and 6 wt% AgNPs, after 21 d on PDMS filled with 3 wt% AgNPs and after 28 d on PDMS filled with 1 wt% and 10 wt% AgNPs were significantly lower (*p* < < 0.0001) than those on glass and PDMS (Fig. 2a).

After 7 days, bacterial densities were significantly lower (*p* < 0.0001) for biofilms that developed on PDMS filled with 6 wt% AgNPs (Fig. 2b). After 14 and 21 days, bacterial densities were significantly lower (*p* < 0.0001) for biofilms on PDMS filled with 1–10 wt% AgNPs. After 28 days, bacterial densities for biofilms on PDMS filled with 1–10 wt% AgNPs were significantly lower (*p* < 0.0001) compared to the densities of biofilms on glass but were similar to those on PDMS (*p* > 0.05).

Significantly lower diatom density was observed for 7-day-old biofilms on PDMS filled with 3 wt% AgNPs compared to that of controls (Fig. 2c, *p* < 0.0001). After 14 days, diatom densities were significantly lower for biofilms on PDMS filled with 1–10 wt% AgNPs (*p* < 0.0001). Furthermore, diatom densities were significantly lower for 21-day-old biofilms on PDMS filled with 1–6 wt% AgNPs (*p* < 0.0001) and for 28-day-old biofilms on PDMS filled with 1 wt% AgNPs (*p* < 0.0001) compared to that of controls.

After 7, 21 and 28 days, no significant differences in chlorophyll *a* (chl *a*) concentrations were observed (Fig. 2d, *p* > 0.05). After 14 days, significantly lower concentrations of chl *a* were detected in biofilms that developed on PDMS filled with 1–6 wt% AgNPs (*p* < 0.0001).

### Bacterial community analysis based on MiSeq sequencing

A total of 286,793 valid reads and 5,231 operational taxonomic units (OTUs) were obtained from 28-day-old biofilms on glass, PDMS and PDMS filled with 1 wt% AgNPs, for which the OTU numbers were 2,261, 1,612 and 1,358, respectively. At a 3% dissimilarity level, Good’s coverage estimations suggested 99.1% to 99.9% of bacterial species were present for all treatments.

**Figure 3:**
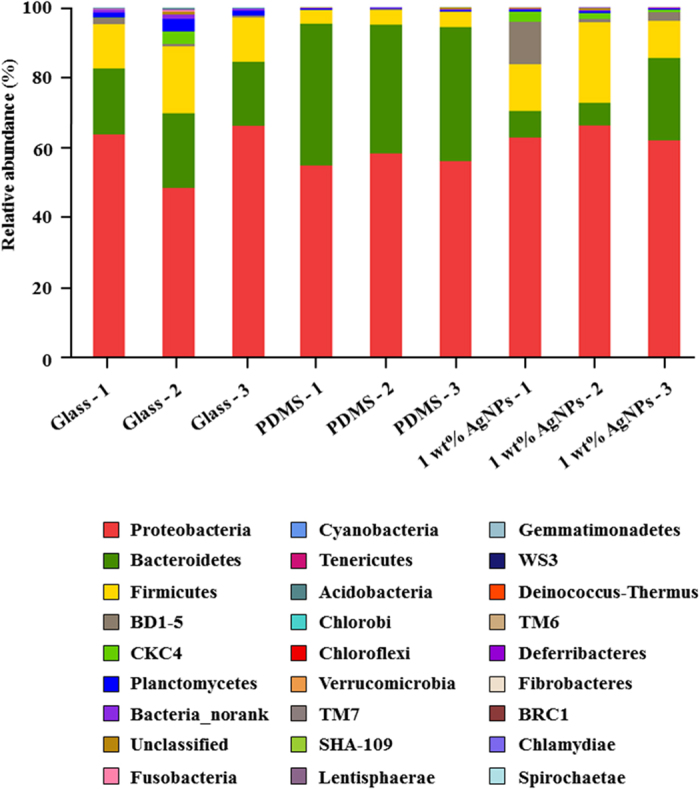
Relative abundance of bacterial phyla in 28-day-old biofilms on glass, PDMS and PDMS filled with 1 wt% AgNPs. Three replicates for each treatment are labelled with the numbers 1, 2 and 3.

At the phylum level, one-way analysis of similarity (ANOSIM) analysis revealed significant differences between the bacterial communities that developed on glass, PDMS and PDMS filled with 1 wt% AgNPs; the R value was 0.802 (*p* < 0.05). Proteobacteria was the most dominant phylum (>45% of all sequences) present in all samples (Fig. 3, Table S1). There was no significant difference in the relative abundance of Proteobacteria between samples (*p* > 0.05, Fig. 3, Table S1). The second most dominant phylum, Bacteroidetes, accounted for 6–40% of the sequences in all biofilm samples, and the relative abundance of Bacteroidetes was significantly higher in biofilms on PDMS than in biofilms on glass and PDMS filled with 1 wt% AgNPs (*p* < 0.05, Table S1). The relative abundance of Firmicutes accounted for 3–23% of the sequences in all samples, and the relative abundance of Firmicutes was significantly lower in biofilms on PDMS than in biofilms on glass and PDMS filled with 1 wt% AgNPs (*p* < 0.05, Table S1). The abundances of bacteria belonging to all other phyla were lower (< 17%).

**Figure 4:**
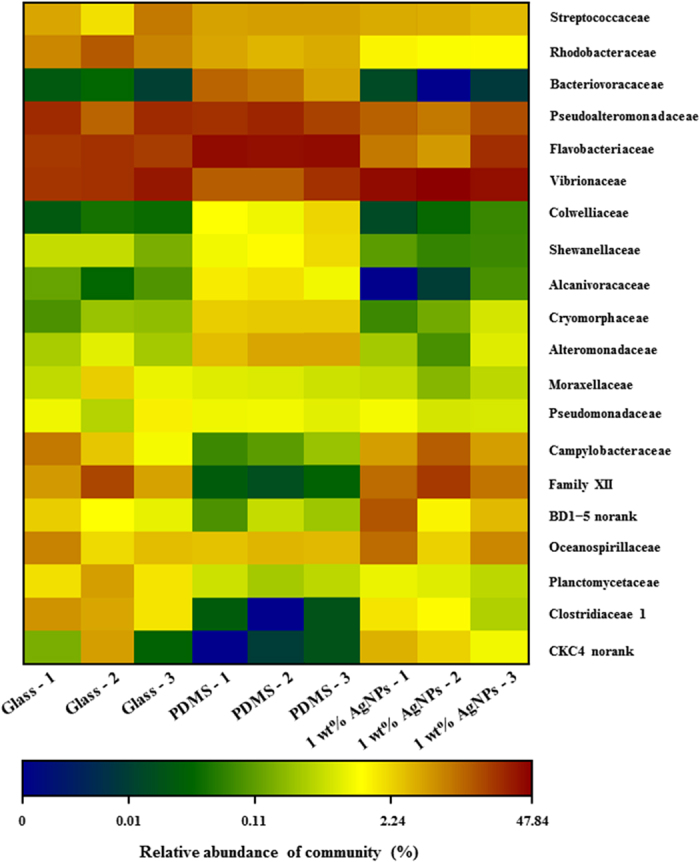
Heatmap revealing the top 20 bacterial families (%) in each biofilm sample on glass, PDMS and PDMS filled with 1 wt% AgNPs. Three replicates for each treatment are labelled with the numbers 1, 2 and 3.

At the family level, significant differences were observed in bacterial communities on different substrata, with an R value of > 0.9 (*p* < 0.05). Pseudoalteromonadaceae, Vibrionaceae and Flavobacteriaceae were the most dominant families present for all three treatments (45–72% of all sequences, Fig. 4, Table S2). No significant difference in the relative abundance of Pseudoalteromonadaceae was observed in bacterial communities in all samples (*p* > 0.05, Fig. 4, Table S2); however, the relative abundance of Vibrionaceae was higher in bacterial communities on PDMS filled with 1 wt% AgNPs than in those on glass and PDMS (*p* < 0.05, Fig. 4, Table S2). Additionally, the relative abundance of Flavobacteriaceae in bacterial communities on PDMS filled with 1 wt% AgNPs was significantly lower than that on PDMS (*p* < 0.05). Based on similarity of percentage (SIMPER) analysis, the families Vibrionaceae, Flavobacteriaceae and BD1–5 explained >5% of the variance between bacterial communities on glass and PDMS filled with 1 wt% AgNPs (Table 1). Vibrionaceae and BD1–5 explained >5% of the variance between marine biofilm communities on PDMS and PDMS filled with 1 wt% AgNPs (Table 1).

**Figure 5:**
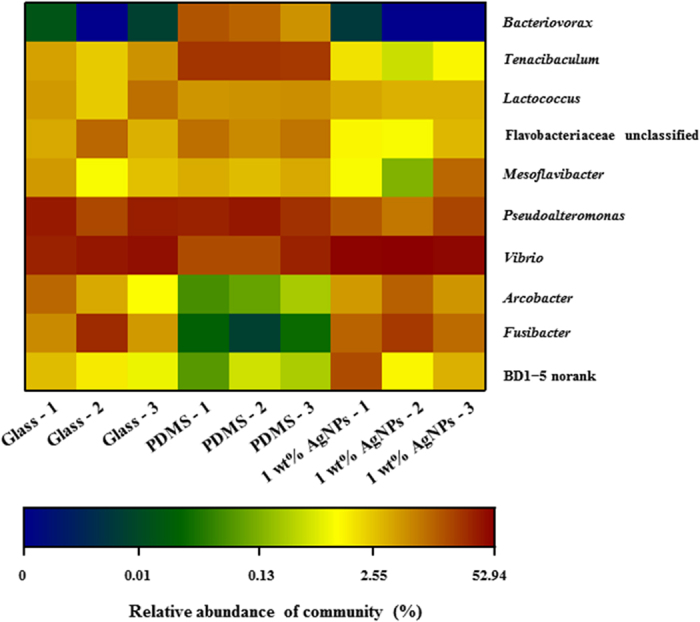
Heatmap revealing the top 10 bacterial genera (%) in each biofilm sample on glass, PDMS and PDMS filled with 1 wt% AgNPs. Three replicates for each treatment are labelled with the numbers 1, 2 and 3.

At the genus level, there was a significant difference in bacterial communities on different substrata, with an R value of > 0.9 (*p* < 0.05). *Vibrio*, *Pseudoalteromonas*, *Fusibacter* and *Tenacibaculum* were the four major genera present in all samples (>40% of all sequences, Fig. 5, Table S3). The average relative abundance of *Vibrio* species increased by more than 0.73-fold and 1.9-fold in bacterial communities that developed on PDMS filled with 1 wt% AgNPs compared to those on glass and PDMS, respectively (*p* < 0.05, Fig. 5, Table S3). Based on SIMPER analysis, the genera *Vibrio* explained >3% of the variance between communities that developed on glass and PDMS filled with 1 wt% AgNPs as well as between communities that developed on PDMS and PDMS filled with 1 wt% AgNPs (Table 2).

**Figure 6:**
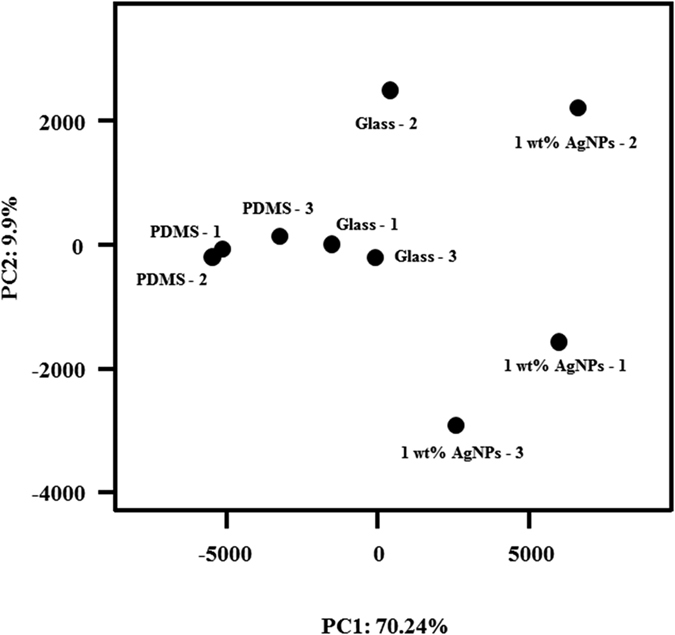
PCA of three biofilm samples on glass, PDMS and PDMS filled with 1 wt% AgNPs. Three replicates for each treatment are labelled with the numbers 1, 2 and 3.

Principal component analysis (PCA) revealed large differences between biofilm communities that developed on glass and PDMS compared to those that developed on PDMS filled with 1 wt% AgNPs (PC1–70.24% of the variance) (Fig. 6).

## Discussion

In marine ecosystems, microbial community succession within a biofilm has been reported^24^,^37^,^38^,^39^,^40^,^41^. However, the mechanisms by which biofilm communities mediate the settlement of marine invertebrate larvae remain poorly constrained^23^,^24^. Additionally, although AgNPs possess excellent anti-biofilm activity^1^,^40^, how AgNPs impact biofilm communities in complex marine environments remains unknown. The present study investigated the impact of AgNPs on biofilm bacterial communities in a marine environment and explored the subsequent effects of biofilms on the settlement of *M. coruscus* plantigrades.

AgNPs are efficient antimicrobial agents that combat the growth of a broad spectrum of bacterial species^1^,^3^. Inbakandan *et al.*^11^ collected marine biofilms that formed at the air-seawater interface on the bottom of the hull of a fishing vessel, isolated 11 bacterial strains and examined the influence of AgNPs on these bacterial species under laboratory conditions. AgNPs exhibited good antibacterial properties, and inhibition depended on the concentration of AgNPs and the bacterial species^11^. Similarly, the experiments in this study revealed the ability of PDMS filled with AgNPs to decrease bacterial densities in marine biofilms after 14 and 21 days compared with controls (glass and PDMS). Thus, in the natural environment, AgNPs exert inhibitory effects on marine bacteria during biofilm formation. The antimicrobial activity of AgNPs may be attributable to the Ag^+^ ions released from the nanoparticles as a result of their exposure to reactive entities generated intracellularly^1^.

Moreover, the results of the present study reveal variations in bacterial communities among biofilms that formed in the presence and absence of AgNPs, indicating colonization by bacterial communities in the marine environment is impacted by AgNPs. This finding is consistent with our hypothesis that AgNPs may affect microbial community formation. According to previous studies, bacterial communities are impacted by many factors, such as surface characteristics, substratum materials and environmental conditions^23^,^39^,^42^,^43^. In addition, variations in biofilm communities on surfaces with and without antifouling properties were recently reviewed^11^. Prior to this study, no study has investigated marine biofilm communities that form on substrata containing AgNPs.

According to our results, Proteobacteria was the most dominant phylum in all marine biofilms, similar to previous investigations^23^,^44^,^45^,^46^. The incorporation of AgNPs reduced the relative abundance of Bacteroidetes (Flavobacteriaceae) and increased the relative abundance of Vibrionaceae (Gammaproteobacteria) bacteria in biofilms. Thus, AgNPs may specifically inhibit Bacteroidetes cell attachment. In contrast, in activated sludge microbial communities, AgNPs increase the abundance of Gammaproteobacteria and Bacteroidetes^47^. These differing results may be explained by the presence of different microbial communities in different environments. Based on further metagenomic analysis at the genus level, AgNPs increased the relative abundance of *Vibrio* and reduced the relative abundances of *Tenacibaculum* and *Shewanella* compared with that of control PDMS (Table 2, Table S3). In previous studies, *Vibrio* spp. inhibited the larval settlement of certain invertebrate larvae, including *H. elegans*^48^,^49^, *Bugula neritina*^48^,^50^, and *B. amphitrite*^51^. However, *Tenacibaculum* spp. and *Shewanella* spp. promote the larval settlement of various invertebrates, including *M. coruscus*^31^ and *B. neritina*^50^. Therefore, the high abundance of *Vibrio* spp. and the lower abundance of *Tenacibaculum* spp. and *Shewanella* spp. in biofilms on PDMS filled with AgNPs may explain the lower mussel settlement observed in the present study.

The antibacterial properties of AgNPs are relatively well documented^52^, while their activities against diatoms have not been well characterized. In the laboratory, AgNPs inhibit the growth of the marine diatom *Thalassiosira pseudonana*^53^. In this study, the densities of marine diatoms in biofilms formed on PDMS filled with AgNPs were lower than controls (glass and PDMS), even after 28 days, suggesting AgNPs effectively inhibit the attachment of diatoms during the formation of natural biofilms in the marine environment. Although the mechanism underlying the anti-diatom action of AgNPs remains unclear, previous studies have described toxic effects of NPs and released Ag^+^ ions on diatoms^53^. AgNPs penetrate the cell wall of the diatom *Cylindrotheca fusiformis* and cause local damage inside the cell without disintegrating the cell wall^54^. Additionally, AgNPs reach the cytoplasm of the diatom *T. pseudonana* and cause toxicity^55^.

In summary, the findings of this study revealed the impact of AgNPs on the formation of marine biofilms, which subsequently inhibited the plantigrade settlement of *M. coruscus*. Thus, AgNPs possess antifouling properties, and it is necessary to perform proper toxicological studies for NPs before their practical application in the marine environment.

## Methods

### Preparation of tested surfaces

AgNPs (CW-Ag-001; particle size: 20 nm; specific surface area: 42 m^2^/g; purity: >99%) were purchased from Shanghai Chaowei Nano Co., Ltd. (China). PDMS, a common fouling released material, was selected as a coating control for our assays. In previous studies, PDMS has been employed to investigate the interactions between substratum properties and the settlement of marine invertebrates^34^,^35^,^36^. As a PDMS support substratum, a glass slide (38 mm × 26 mm, AS ONE Shanghai Corporation, Shanghai, China) was used as an additional control and conveniently submerged during biofilm formation. PDMS (Sylgard 184; Dow Corning, Shanghai, China) was made according to the method of Carl *et al.*^34^. AgNPs were incorporated into the PDMS matrix (PDMS filled with AgNPs) at concentrations of 0 (PDMS only), 1, 3, 6 and 10 weight% (wt%). The bubbles created from mixing PDMS with AgNPs were removed with a vacuum desiccator. Then, the mixture was poured onto a glass slide and cured for up to 10 h in a hot oven at 90 °C.

### Development of natural biofilms

Coated substrata were randomly placed on PVC holders and submerged vertically at a depth of approximately 1.0 m at Gouqi Island (122°77′E; 30°72′N), Zhoushan, China for 7, 14, 21 and 28 days in August 2013. Biofilm samples were collected and transported on ice to Shanghai Ocean University, and macrofouling organisms were removed.

### Biofilm dry weight

The dry weight of biofilms was evaluated following the method of Bao *et al.*^33^. In brief, biofilms from six treatment replicates (n = 24) were scraped off substrata surfaces into 100-ml beakers containing autoclaved filter seawater (AFSW). An additional 50 ml of 0.22-μm AFSW was used to wash the biofilms attached to the beakers. Then, the suspensions were filtered using a pre-weighed Whatman GF/C filter (pore size: 1.2 μm, Whatman International Ltd, Maidstone, England). Each GF/C filter paper was dried at 80 °C for 48 h. The dry weight of each biofilm sample was calculated based on the weight difference between the weight of the filter before and after filtration.

### Bacterial and diatom density

Bacterial densities were determined according to the method of Yang *et al.*^56^. Natural biofilms from each treatment (n = 24) were separately scraped off using sterile glass slides, suspended in AFSW, and fixed in formalin (final concentration = 5%). Suspensions containing bacteria and diatoms were vortexed for 60 s to ensure the homogeneous distribution of microorganisms before counting cell densities. Bacteria were stained with 0.1% acridine orange (Sigma Chemical Co., St Louis, Mo) solution. The suspended bacteria were filtered and collected on polycarbonate Nucleopore filters (pore size: 0.2 μm, Whatman 4.9 cm^2^, Whatman International Ltd, Maidstone, England). The bacterial densities of each stained sample (n = 3) were immediately determined with an Olympus BX51 epifluorescence microscope (Olympus, Tokyo, Japan) at 1000× magnification. The cell densities of suspended diatoms (n = 3) were evaluated using a hemocytometer under an Olympus BX51 epifluorescence microscope (Olympus, Tokyo, Japan) with 200× magnification. Ten random fields of view for each biofilm sample were counted, and the mean densities of bacteria and diatoms were calculated.

### Chlorophyll *a* (chl *a*) concentration in biofilms

Biofilms from each treatment (n = 24) were scraped, filtered and preserved at −20 °C. A 90% acetone solution was used to extract chl *a* at 4 °C for 14 h in the dark. After centrifugation, chl *a* concentrations in the supernatants were evaluated spectrophotometrically (UNIC 2100 spectrophotometer, UNIC (Shanghai) Instruments, Shanghai, China) according to the method of Wang *et al.*^29^. Six independent glass slides were examined for each treatment.

### MiSeq sequencing analysis of bacterial communities

Bacterial DNA was extracted from each 28-day-old biofilm sample (glass, PDMS, PDMS filled with 1 wt% AgNPs) with 3 biological replicates according to Li *et al.*^45^. The V4-V5 region of the 16 S rRNA gene was amplified using previously described primers and condition^45^. PCR products were extracted, purified and quantified following Li *et al.*^45^. The purified amplicons were pooled in equimolar concentrations and sequenced on the Illumina MiSeq platform at Majorbio Bio-Pharm Technology Co., Ltd., Shanghai, China. The generated metagenomic data were deposited in the NCBI sequence read archive (accession number: SRP 048906).

### Mussel settlement assay

Mussel plantigrades were supplied by the Institute of Marine Science and Technology in Zhoushan, China. The culture conditions were as described by Yang *et al.*^57^. A plantigrade settlement assay was conducted in Petri dishes containing 20 ml of AFSW and one biofilm-covered (7, 14, 21 and 28 days) substratum (glass [control], PDMS [control], 1 wt% AgNPs, 3 wt% AgNPs, 6 wt% AgNPs and 10 wt% AgNPs). Ten plantigrades were added into each Petri dish (AS ONE Shanghai Corporation, Shanghai, China). The number of settled plantigrades was counted after 12 h and later calculated as a percentage. The effects of substrata without biofilms (glass, PDMS, PDMS filled with AgNPs) on plantigrade settlement were examined. Plantigrades attached to the coating via byssal threads were considered settled. The mussel settlement assay was performed according to the method of Yang *et al.*^57^. Six replicates were examined for each biofilm and non-biofilm treatment.

### Statistical and bioinformatics analysis

To ensure data normality, the percentages of biofilm-inducing activity were arcsine-square root transformed. The data were analyzed using JMP^TM^ software (SAS Institute (Shanghai) Co., LTD, Shanghai, China). A *p* value of 0.05 (*p* < 0.05) was considered significant. Settlement rate, dry weight, chl *a* and bacteria and diatom density analyses were conducted by performing Kruskal-Wallis analysis of variance followed by the Steel-Dwass All Pairs test.

ANOSIM (PRIMER 6)^58^ was performed with substratum as the factor. Specifically, ANOSIM was conducted to compare similarities in bacterial species composition between substrata with and without AgNPs. ANOSIM computes a test statistic (R), where R = 1 if all replicates within a treatment are more similar to each other than any replicates from different treatments. R is approximately zero if the null hypothesis that similarities between and within treatments are the same is true. ANOSIM analysis was performed for each biofilm sample (n = 3). SIMPER analysis (Primer 6 software; Primer-E Ltd) was performed to compare the major species contributing to the dissimilarity between bacterial communities that developed on substrata with and without AgNPs.

Raw fastq data were analyzed using QIIME (version 1.17)^59^. The processing steps were as follows. The raw mate-paired fastq files were demultiplexed and quality-filtered as described previously^45^. The UPARSE pipeline (version 7.1 http://drive5.com/uparse/) was applied to choose the OTUs with a similarity threshold of 97%. The phylogenetic affiliation of each 16 S rRNA gene sequence was evaluated with RDP classifier (http://rdp.cme.msu.edu/) against the Silva (SSU115) 16 S rRNA database using a confidence threshold of 70%^60^.

## Additional Information

**How to cite this article**: Yang, J.-L. *et al.* Silver Nanoparticles Impact Biofilm Communities and Mussel Settlement. *Sci. Rep.* **6**, 37406; doi: 10.1038/srep37406 (2016).

**Publisher’s note:** Springer Nature remains neutral with regard to jurisdictional claims in published maps and institutional affiliations.

## Supplementary Material

Supplementary Information

## Figures and Tables

**Table 1:** SIMPER analysis showing the % contribution of specific bacterial families to the total dissimilarity among bacterial communities that developed on glass, PDMS, and PDMS filled with 1 wt% AgNPs.

Family	Comparison with PDMS filled with 1 wt% AgNPs	
	Glass	PDMS
Vibrionaceae	6.45	7.58
Flavobacteriaceae		6.72
BD1-5	5.28	5.43
Others	88.27	80.27

Note: The contributions are averaged over all significant pair-wise treatment comparisons. Bacteria that contributed less than 5% are not shown.

**Table 2:** SIMPER analysis showing the % contribution of specific bacterial genera to the total dissimilarity among bacterial communities that developed on glass, PDMS, and PDMS filled with 1 wt% AgNPs.

Genus	Comparison with PDMS filled with 1 wt% AgNPs	
	Glass	PDMS
*Vibrio*	3.24	4.47
*Tenacibaculum*	1.22	4.59
*Shewanella*		1.24
Others	95.54	89.70

Note: The contributions are averaged over all significant pair-wise treatment comparisons.
