# Supplementary material for: Silver Nanoparticles Impact Biofilm Communities and Mussel Settlement
Source: Sci Rep. 2016 Nov 21;6:37406. doi: 10.1038/srep37406 (PMC5116650; doi:10.1038/srep37406)
Supplement: Supplementary Information [file srep37406-s1.pdf]

# Silver Nanoparticles Impact Biofilm Communities and Mussel Settlement

Jin-Long Yang[1,2,3*], Yi-Feng Li[1*], Xiao Liang[1*], Xing-Pan Guo[1], De-Wen Ding[2], Demin Zhang[3], Shuxue Zhou[4], Wei-Yang Bao[5], Nikoleta Bellou[6], Sergey Dobretsov[7,8]

[1] Key Laboratory of Exploration and Utilization of Aquatic Genetic Resources, Shanghai Ocean University, Ministry of Education, China

[2] Marine Ecology Research Center, The First Institute of Oceanography, State Oceanic Administration, Qingdao, China

[3] Collaborative Innovation Center for Zhejiang Marine High-efficiency and Healthy Aquaculture, Ningbo, China

[4] Department of Materials Science, State Key Laboratory of Molecular Engineering of Polymers, Advanced Coatings Research Center of Ministry of Education of China, Fudan University, Shanghai, China

[5] Institute of Marine Science and Technology, Yangzhou University, Yangzhou, China

[6] Hellenic Centre for Marine Research, Institute of Oceanography, Athens, Greece

[7] Department of Marine Science and Fisheries, College of Agricultural and Marine Sciences, Sultan Qaboos University, Muscat, Oman

[8] Center of Excellence in Marine Biotechnology, Sultan Qaboos University, Muscat, Oman

Corresponding authors:

Jin-Long Yang[1,2,3], No. 999 Hucheng Huan Road, Shanghai, 201306, China, e-mail address: jlyang@shou.edu.cn

Wei-Yang Bao[5], No. 88 South University Ave., Yangzhou, Jiangsu, 225009, China, e-mail address: baowy@yzu.edu.cn

[*]These authors contributed equally.

## Supplementary Tables

**Table S1.** Relative abundance (in %) of the sequences from biofilms on three substrata assigned to different phyla.

| **Phyla** | **Glass-1** | **Glass-2** | **Glass-3** | **PDMS-1** | **PDMS-2** | **PDMS-3** | **1 wt% AgNPs-1** | **1 wt% AgNPs-2** | **1 wt% AgNPs-3** |
|---|---|---|---|---|---|---|---|---|---|
| Acidobacteria | 0.07 | 0.16 | 0.05 | 0.02 | 0.01 | 0.03 | 0.01 | 0.02 | 0.01 |
| Armatimonadetes | 0.00 | 0.00 | 0.00 | 0.00 | 0.00 | 0.00 | 0.00 | 0.00 | 0.00 |
| BD1-5 | 1.81 | 0.72 | 0.56 | 0.05 | 0.33 | 0.18 | 12.14 | 0.98 | 2.53 |
| Bacteria_norank | 0.82 | 1.27 | 0.55 | 0.28 | 0.20 | 0.30 | 0.21 | 0.21 | 0.16 |
| Bacteria_unclassified | 0.03 | 0.85 | 0.05 | 0.02 | 0.01 | 0.49 | 0.36 | 0.64 | 0.07 |
| Bacteroidetes | 18.87 | 21.35 | 18.30 | 40.43 | 36.75 | 38.40 | 7.59 | 6.47 | 23.52 |
| CKC4 | 0.10 | 3.50 | 0.01 | 0.00 | 0.00 | 0.01 | 2.90 | 1.72 | 0.67 |
| Candidate_division_BRC1 | 0.00 | 0.00 | 0.00 | 0.00 | 0.00 | 0.00 | 0.00 | 0.00 | 0.00 |
| Candidate_division_OD1 | 0.00 | 0.00 | 0.00 | 0.00 | 0.00 | 0.00 | 0.00 | 0.00 | 0.00 |
| Candidate_division_OP11 | 0.00 | 0.00 | 0.00 | 0.00 | 0.00 | 0.00 | 0.00 | 0.00 | 0.00 |
| Candidate_division_OP3 | 0.00 | 0.00 | 0.00 | 0.00 | 0.00 | 0.00 | 0.00 | 0.00 | 0.00 |
| Candidate_division_OP8 | 0.00 | 0.00 | 0.00 | 0.00 | 0.00 | 0.00 | 0.00 | 0.00 | 0.00 |
| Candidate_division_SR1 | 0.00 | 0.00 | 0.00 | 0.00 | 0.00 | 0.00 | 0.00 | 0.00 | 0.00 |
| Candidate_division_TM7 | 0.03 | 0.06 | 0.01 | 0.00 | 0.00 | 0.01 | 0.00 | 0.00 | 0.01 |
| Candidate_division_WS3 | 0.01 | 0.01 | 0.00 | 0.01 | 0.00 | 0.00 | 0.00 | 0.00 | 0.00 |
| Chlamydiae | 0.00 | 0.00 | 0.00 | 0.00 | 0.00 | 0.00 | 0.00 | 0.00 | 0.00 |
| Chlorobi | 0.03 | 0.05 | 0.02 | 0.03 | 0.02 | 0.01 | 0.01 | 0.01 | 0.02 |
| Chloroflexi | 0.01 | 0.09 | 0.02 | 0.01 | 0.00 | 0.00 | 0.01 | 0.01 | 0.00 |
| Cyanobacteria | 0.13 | 0.20 | 0.10 | 0.03 | 0.02 | 0.03 | 0.02 | 0.02 | 0.01 |
| Deferribacteres | 0.00 | 0.00 | 0.00 | 0.00 | 0.00 | 0.00 | 0.00 | 0.00 | 0.00 |
| Deinococcus-Thermus | 0.00 | 0.01 | 0.00 | 0.00 | 0.00 | 0.00 | 0.00 | 0.00 | 0.00 |
| Elusimicrobia | 0.00 | 0.00 | 0.00 | 0.00 | 0.00 | 0.00 | 0.00 | 0.00 | 0.00 |
| Fibrobacteres | 0.00 | 0.00 | 0.01 | 0.00 | 0.00 | 0.00 | 0.00 | 0.00 | 0.00 |
| Firmicutes | 12.75 | 19.09 | 12.73 | 3.93 | 4.23 | 4.26 | 13.33 | 22.93 | 10.59 |
| Fusobacteria | 0.20 | 0.38 | 0.02 | 0.01 | 0.01 | 0.01 | 0.02 | 0.01 | 0.01 |

| | | | | | | | | | |
|---|---|---|---|---|---|---|---|---|---|
| Gemmatimonadetes | 0.00 | 0.02 | 0.00 | 0.00 | 0.01 | 0.00 | 0.00 | 0.00 | 0.00 |
| Lentisphaerae | 0.00 | 0.02 | 0.01 | 0.00 | 0.00 | 0.00 | 0.00 | 0.00 | 0.01 |
| Nitrospirae | 0.00 | 0.00 | 0.00 | 0.00 | 0.00 | 0.00 | 0.00 | 0.00 | 0.00 |
| Planctomycetes | 1.34 | 3.58 | 1.30 | 0.39 | 0.24 | 0.31 | 0.61 | 0.52 | 0.29 |
| Proteobacteria | 63.65 | 48.48 | 66.19 | 54.79 | 58.16 | 55.95 | 62.71 | 66.24 | 61.97 |
| SHA-109 | 0.02 | 0.01 | 0.01 | 0.00 | 0.00 | 0.00 | 0.02 | 0.00 | 0.01 |
| Spirochaetae | 0.00 | 0.00 | 0.00 | 0.00 | 0.00 | 0.00 | 0.00 | 0.00 | 0.00 |
| Synergistetes | 0.00 | 0.00 | 0.00 | 0.00 | 0.00 | 0.00 | 0.00 | 0.00 | 0.00 |
| TM6 | 0.00 | 0.00 | 0.00 | 0.00 | 0.00 | 0.00 | 0.00 | 0.00 | 0.00 |
| Tenericutes | 0.07 | 0.09 | 0.02 | 0.00 | 0.00 | 0.00 | 0.05 | 0.19 | 0.13 |
| Verrucomicrobia | 0.06 | 0.04 | 0.01 | 0.01 | 0.00 | 0.00 | 0.01 | 0.02 | 0.00 |

**Table S2.** Relative abundance (in %) of the sequences from biofilms on glass, PDMS and PDMS filled with 1 wt% AgNPs assigned to different families.

| **Family** | **Glass-1** | **Glass-2** | **Glass-3** | **PDMS-1** | **PDMS-2** | **PDMS-3** | **1 wt% AgNPs-1** | **1 wt% AgNPs-2** | **1 wt% AgNPs-3** |
|---|---|---|---|---|---|---|---|---|---|
| 0319-6G20 | 0.00 | 0.00 | 0.00 | 0.00 | 0.00 | 0.00 | 0.00 | 0.00 | 0.00 |
| 288-2 | 0.00 | 0.00 | 0.00 | 0.00 | 0.00 | 0.00 | 0.00 | 0.00 | 0.00 |
| 34P16_norank | 0.00 | 0.00 | 0.00 | 0.00 | 0.00 | 0.00 | 0.00 | 0.00 | 0.00 |
| 43F-1404R_norank | 0.00 | 0.00 | 0.00 | 0.00 | 0.00 | 0.00 | 0.00 | 0.00 | 0.00 |
| ABS-19 | 0.00 | 0.00 | 0.00 | 0.00 | 0.00 | 0.00 | 0.00 | 0.00 | 0.00 |
| AEGEAN-245_norank | 0.00 | 0.00 | 0.00 | 0.00 | 0.00 | 0.00 | 0.00 | 0.00 | 0.00 |
| AKIW1012 | 0.00 | 0.00 | 0.00 | 0.00 | 0.00 | 0.00 | 0.00 | 0.00 | 0.00 |
| AT-s3-28_norank | 0.00 | 0.00 | 0.00 | 0.00 | 0.00 | 0.00 | 0.00 | 0.00 | 0.00 |
| Acetobacteraceae | 0.00 | 0.00 | 0.00 | 0.00 | 0.00 | 0.00 | 0.00 | 0.00 | 0.00 |
| Acholeplasmataceae | 0.07 | 0.01 | 0.00 | 0.00 | 0.00 | 0.00 | 0.02 | 0.04 | 0.07 |
| Acidaminococcaceae | 0.00 | 0.00 | 0.00 | 0.00 | 0.00 | 0.00 | 0.00 | 0.00 | 0.00 |
| Acidimicrobiaceae | 0.26 | 0.69 | 0.14 | 0.05 | 0.02 | 0.03 | 0.06 | 0.02 | 0.01 |
| Acidimicrobiales_unclassified | 0.00 | 0.00 | 0.00 | 0.00 | 0.00 | 0.00 | 0.00 | 0.00 | 0.00 |
| Acidimicrobiales_uncultured | 0.07 | 0.11 | 0.02 | 0.01 | 0.00 | 0.00 | 0.02 | 0.01 | 0.00 |
| Acidobacteria_norank | 0.01 | 0.01 | 0.00 | 0.00 | 0.00 | 0.00 | 0.00 | 0.00 | 0.00 |
| Actinobacteria_norank | 0.00 | 0.00 | 0.00 | 0.00 | 0.00 | 0.00 | 0.00 | 0.00 | 0.00 |
| Adriatic90_norank | 0.00 | 0.00 | 0.00 | 0.00 | 0.00 | 0.00 | 0.00 | 0.00 | 0.00 |

| | | | | | | | | | |
|---|---|---|---|---|---|---|---|---|---|
| Aerococcaceae | 0.00 | 0.00 | 0.01 | 0.00 | 0.00 | 0.00 | 0.00 | 0.00 | 0.00 |
| Aeromonadaceae | 0.00 | 0.35 | 0.01 | 0.00 | 0.00 | 0.00 | 0.07 | 0.05 | 0.15 |
| Alcaligenaceae | 0.00 | 0.00 | 0.00 | 0.00 | 0.00 | 0.00 | 0.00 | 0.00 | 0.00 |
| Alcanivoracaceae | 0.08 | 0.01 | 0.05 | 1.16 | 1.36 | 0.68 | 0.00 | 0.00 | 0.05 |
| Alicyclobacillaceae | 0.01 | 0.01 | 0.01 | 0.01 | 0.01 | 0.01 | 0.01 | 0.00 | 0.00 |
| Alphaproteobacteria_unclassified | 0.02 | 0.01 | 0.00 | 0.00 | 0.00 | 0.01 | 0.00 | 0.00 | 0.00 |
| Alteromonadaceae | 0.21 | 0.49 | 0.20 | 2.36 | 3.34 | 3.35 | 0.21 | 0.05 | 0.51 |
| Alteromonadales_unclassified | 0.01 | 0.01 | 0.03 | 0.00 | 0.00 | 0.00 | 0.00 | 0.00 | 0.00 |
| Anaerolineaceae | 0.01 | 0.04 | 0.01 | 0.01 | 0.00 | 0.00 | 0.00 | 0.01 | 0.00 |
| Ardenticatenales_norank | 0.00 | 0.00 | 0.00 | 0.00 | 0.00 | 0.00 | 0.00 | 0.00 | 0.00 |
| Ardenticatenia_uncultured | 0.00 | 0.04 | 0.01 | 0.00 | 0.00 | 0.00 | 0.00 | 0.00 | 0.00 |
| Armatimonadetes_norank | 0.00 | 0.00 | 0.00 | 0.00 | 0.00 | 0.00 | 0.00 | 0.00 | 0.00 |
| Aurantimonadaceae | 0.00 | 0.00 | 0.00 | 0.00 | 0.00 | 0.00 | 0.00 | 0.00 | 0.00 |
| BD1-5_norank | 1.81 | 0.72 | 0.56 | 0.05 | 0.33 | 0.18 | 12.14 | 0.98 | 2.53 |
| BD2-11_terrestrial_group_norank | 0.00 | 0.00 | 0.00 | 0.00 | 0.00 | 0.00 | 0.00 | 0.00 | 0.00 |
| BD2-2_norank | 0.00 | 0.01 | 0.00 | 0.01 | 0.00 | 0.00 | 0.00 | 0.00 | 0.00 |
| BD7-8_marine_group_norank | 0.00 | 0.00 | 0.00 | 0.00 | 0.00 | 0.00 | 0.00 | 0.00 | 0.00 |
| BG.g7_norank | 0.00 | 0.00 | 0.00 | 0.00 | 0.00 | 0.00 | 0.00 | 0.00 | 0.00 |
| BSV26 | 0.00 | 0.00 | 0.00 | 0.00 | 0.00 | 0.00 | 0.00 | 0.00 | 0.00 |
| Bacillaceae | 0.09 | 0.02 | 0.09 | 0.06 | 0.05 | 0.07 | 0.04 | 0.06 | 0.07 |
| Bacteria_unclassified | 0.03 | 0.85 | 0.05 | 0.02 | 0.01 | 0.49 | 0.36 | 0.64 | 0.07 |
| Bacteriovoracaceae | 0.01 | 0.01 | 0.00 | 9.63 | 7.43 | 3.53 | 0.01 | 0.00 | 0.00 |

| | | | | | | | | | |
|---|---|---|---|---|---|---|---|---|---|
| Bacteroidaceae | 0.00 | 0.08 | 0.00 | 0.00 | 0.00 | 0.00 | 0.06 | 0.36 | 0.00 |
| Bacteroidales_unclassified | 0.00 | 0.02 | 0.00 | 0.00 | 0.00 | 0.00 | 0.01 | 0.01 | 0.00 |
| Bacteroidetes_unclassified | 0.00 | 0.01 | 0.01 | 0.05 | 0.08 | 0.02 | 0.00 | 0.00 | 0.00 |
| Bdellovibrionaceae | 0.00 | 0.00 | 0.00 | 0.00 | 0.00 | 0.00 | 0.00 | 0.00 | 0.00 |
| Beutenbergiaceae | 0.00 | 0.01 | 0.00 | 0.00 | 0.00 | 0.00 | 0.00 | 0.00 | 0.00 |
| Bifidobacteriaceae | 0.00 | 0.01 | 0.02 | 0.00 | 0.00 | 0.01 | 0.00 | 0.00 | 0.00 |
| Brevibacteriaceae | 0.00 | 0.00 | 0.00 | 0.00 | 0.00 | 0.00 | 0.00 | 0.00 | 0.00 |
| Brucellaceae | 0.00 | 0.00 | 0.00 | 0.00 | 0.00 | 0.00 | 0.00 | 0.00 | 0.00 |
| Burkholderiaceae | 0.01 | 0.00 | 0.00 | 0.00 | 0.00 | 0.00 | 0.00 | 0.00 | 0.00 |
| CA002 | 0.00 | 0.00 | 0.00 | 0.00 | 0.00 | 0.00 | 0.00 | 0.00 | 0.00 |
| CKC4_norank | 0.10 | 3.50 | 0.01 | 0.00 | 0.00 | 0.01 | 2.90 | 1.72 | 0.67 |
| Caenarcaniphilales_norank | 0.00 | 0.00 | 0.00 | 0.00 | 0.00 | 0.00 | 0.00 | 0.00 | 0.00 |
| Caldilineaceae | 0.00 | 0.01 | 0.00 | 0.00 | 0.00 | 0.00 | 0.00 | 0.00 | 0.00 |
| Campylobacteraceae | 6.72 | 1.97 | 0.71 | 0.04 | 0.07 | 0.16 | 3.75 | 10.16 | 3.90 |
| Candidate_division_BRC1_norank | 0.00 | 0.00 | 0.00 | 0.00 | 0.00 | 0.00 | 0.00 | 0.00 | 0.00 |
| Candidate_division_OD1_norank | 0.00 | 0.00 | 0.00 | 0.00 | 0.00 | 0.00 | 0.00 | 0.00 | 0.00 |
| Candidate_division_OP11_norank | 0.00 | 0.00 | 0.00 | 0.00 | 0.00 | 0.00 | 0.00 | 0.00 | 0.00 |
| Candidate_division_OP3_norank | 0.00 | 0.00 | 0.00 | 0.00 | 0.00 | 0.00 | 0.00 | 0.00 | 0.00 |
| Candidate_division_OP8_norank | 0.00 | 0.00 | 0.00 | 0.00 | 0.00 | 0.00 | 0.00 | 0.00 | 0.00 |
| Candidate_division_SR1_norank | 0.00 | 0.00 | 0.00 | 0.00 | 0.00 | 0.00 | 0.00 | 0.00 | 0.00 |
| Candidate_division_TM7_norank | 0.03 | 0.06 | 0.01 | 0.00 | 0.00 | 0.01 | 0.00 | 0.00 | 0.01 |
| Candidate_division_TM7_unclassified | 0.00 | 0.00 | 0.00 | 0.00 | 0.00 | 0.00 | 0.00 | 0.00 | 0.00 |

| | | | | | | | | | |
|---|---|---|---|---|---|---|---|---|---|
| Candidate_division_WS3_norank | 0.01 | 0.01 | 0.00 | 0.01 | 0.00 | 0.00 | 0.00 | 0.00 | 0.00 |
| Carnobacteriaceae | 0.06 | 0.01 | 0.08 | 0.01 | 0.03 | 0.03 | 0.04 | 0.02 | 0.03 |
| Caulobacteraceae | 0.00 | 0.00 | 0.00 | 0.00 | 0.00 | 0.00 | 0.00 | 0.00 | 0.00 |
| Chitinophagaceae | 0.01 | 0.02 | 0.00 | 0.00 | 0.01 | 0.00 | 0.00 | 0.00 | 0.01 |
| Chlamydiaceae | 0.00 | 0.00 | 0.00 | 0.00 | 0.00 | 0.00 | 0.00 | 0.00 | 0.00 |
| Chlamydiales_unclassified | 0.00 | 0.00 | 0.00 | 0.00 | 0.00 | 0.00 | 0.00 | 0.00 | 0.00 |
| Chloroflexi_uncultured | 0.00 | 0.00 | 0.00 | 0.00 | 0.00 | 0.00 | 0.00 | 0.00 | 0.00 |
| Chromatiaceae | 0.00 | 0.00 | 0.00 | 0.00 | 0.00 | 0.00 | 0.00 | 0.00 | 0.00 |
| Clostridiaceae_1 | 4.41 | 3.08 | 1.24 | 0.01 | 0.00 | 0.01 | 1.31 | 0.88 | 0.24 |
| Clostridiaceae_2 | 0.01 | 0.00 | 0.00 | 0.00 | 0.00 | 0.00 | 0.00 | 0.00 | 0.00 |
| Clostridiaceae_3 | 0.08 | 0.06 | 0.22 | 0.00 | 0.00 | 0.00 | 0.02 | 0.03 | 0.00 |
| Clostridiales_clostridiales_incertae_sedis | 0.00 | 0.00 | 0.00 | 0.00 | 0.00 | 0.00 | 0.00 | 0.00 | 0.00 |
| Clostridiales_unclassified | 0.02 | 0.05 | 0.01 | 0.00 | 0.00 | 0.00 | 0.11 | 0.02 | 0.02 |
| Colwelliaceae | 0.01 | 0.02 | 0.02 | 0.80 | 0.65 | 1.59 | 0.01 | 0.02 | 0.04 |
| Comamonadaceae | 0.01 | 0.00 | 0.01 | 0.01 | 0.01 | 0.00 | 0.01 | 0.01 | 0.00 |
| Corynebacteriaceae | 0.00 | 0.00 | 0.01 | 0.00 | 0.00 | 0.00 | 0.00 | 0.01 | 0.00 |
| Coxiellaceae | 0.01 | 0.03 | 0.02 | 0.00 | 0.01 | 0.01 | 0.01 | 0.00 | 0.01 |
| Crenotrichaceae | 0.00 | 0.00 | 0.00 | 0.00 | 0.00 | 0.00 | 0.00 | 0.00 | 0.00 |
| Cryomorphaceae | 0.05 | 0.16 | 0.15 | 1.89 | 1.95 | 1.91 | 0.04 | 0.09 | 0.42 |
| Cyanobacteria_norank | 0.06 | 0.08 | 0.04 | 0.01 | 0.02 | 0.03 | 0.01 | 0.01 | 0.00 |
| Cyclobacteriaceae | 0.01 | 0.00 | 0.00 | 0.00 | 0.00 | 0.00 | 0.00 | 0.00 | 0.00 |
| Cystobacteraceae | 0.00 | 0.00 | 0.00 | 0.00 | 0.00 | 0.00 | 0.00 | 0.00 | 0.00 |

| | | | | | | | | | |
|---|---|---|---|---|---|---|---|---|---|
| Cytophagaceae | 0.52 | 0.62 | 0.60 | 0.00 | 0.02 | 0.01 | 0.16 | 0.50 | 0.06 |
| Cytophagales_unclassified | 0.00 | 0.01 | 0.00 | 0.00 | 0.00 | 0.00 | 0.00 | 0.00 | 0.00 |
| DB1-14_norank | 0.14 | 0.13 | 0.12 | 0.01 | 0.03 | 0.07 | 0.05 | 0.05 | 0.03 |
| DEV007 | 0.02 | 0.01 | 0.00 | 0.00 | 0.00 | 0.00 | 0.00 | 0.00 | 0.00 |
| Deferribacterales_deferribacterales_incertae_sedis | 0.00 | 0.00 | 0.00 | 0.00 | 0.00 | 0.00 | 0.00 | 0.00 | 0.00 |
| Defluviitaleaceae | 0.32 | 0.04 | 0.00 | 0.00 | 0.00 | 0.00 | 0.08 | 0.04 | 0.00 |
| Deinococcaceae | 0.00 | 0.00 | 0.00 | 0.00 | 0.00 | 0.00 | 0.00 | 0.00 | 0.00 |
| Deltaproteobacteria_unclassified | 0.00 | 0.00 | 0.00 | 0.00 | 0.00 | 0.00 | 0.00 | 0.00 | 0.00 |
| Desulfarculaceae | 0.00 | 0.00 | 0.00 | 0.00 | 0.00 | 0.00 | 0.00 | 0.00 | 0.00 |
| Desulfobacteraceae | 0.00 | 0.01 | 0.00 | 0.01 | 0.00 | 0.00 | 0.00 | 0.00 | 0.00 |
| Desulfobulbaceae | 0.04 | 0.09 | 0.05 | 0.01 | 0.00 | 0.02 | 0.00 | 0.00 | 0.01 |
| Dietziaceae | 0.00 | 0.00 | 0.00 | 0.01 | 0.00 | 0.00 | 0.00 | 0.00 | 0.00 |
| E01-9C-26_marine_group_norank | 0.01 | 0.01 | 0.00 | 0.00 | 0.00 | 0.00 | 0.01 | 0.00 | 0.00 |
| EC3_norank | 0.02 | 0.06 | 0.01 | 0.00 | 0.01 | 0.00 | 0.01 | 0.01 | 0.00 |
| EV818SWSAP88 | 0.00 | 0.01 | 0.00 | 0.00 | 0.00 | 0.00 | 0.00 | 0.00 | 0.00 |
| Ectothiorhodospiraceae | 0.01 | 0.03 | 0.01 | 0.01 | 0.00 | 0.01 | 0.00 | 0.00 | 0.00 |
| Elev-16S-1332 | 0.00 | 0.00 | 0.00 | 0.00 | 0.00 | 0.00 | 0.00 | 0.00 | 0.00 |
| Enterobacteriaceae | 0.08 | 0.05 | 0.08 | 0.07 | 0.05 | 0.05 | 0.08 | 0.02 | 0.02 |
| Enterococcaceae | 0.05 | 0.02 | 0.07 | 0.01 | 0.03 | 0.04 | 0.03 | 0.05 | 0.04 |
| Erysipelotrichaceae | 0.00 | 0.05 | 0.00 | 0.00 | 0.00 | 0.00 | 0.01 | 0.01 | 0.00 |
| Erythrobacteraceae | 0.57 | 0.52 | 0.37 | 0.29 | 0.25 | 0.22 | 0.13 | 0.06 | 0.05 |
| FS117-23B-02_norank | 0.00 | 0.00 | 0.00 | 0.00 | 0.00 | 0.00 | 0.00 | 0.00 | 0.00 |

| | | | | | | | | | |
|---|---|---|---|---|---|---|---|---|---|
| Family_XI | 0.00 | 0.06 | 0.00 | 0.00 | 0.00 | 0.00 | 0.00 | 0.01 | 0.01 |
| Family_XII | 3.86 | 13.97 | 3.57 | 0.01 | 0.01 | 0.01 | 8.26 | 18.22 | 7.44 |
| Family_XIII | 0.04 | 0.01 | 0.00 | 0.00 | 0.00 | 0.00 | 0.00 | 0.00 | 0.00 |
| Ferrimonadaceae | 0.06 | 0.02 | 0.01 | 0.02 | 0.01 | 0.06 | 0.01 | 0.04 | 0.03 |
| Fibrobacteraceae | 0.00 | 0.00 | 0.01 | 0.00 | 0.00 | 0.00 | 0.00 | 0.00 | 0.00 |
| Flammeovirgaceae | 0.10 | 0.27 | 0.24 | 0.04 | 0.03 | 0.05 | 0.01 | 0.05 | 0.07 |
| Flavobacteriaceae | 17.67 | 18.29 | 16.69 | 38.09 | 34.44 | 36.13 | 7.04 | 4.05 | 22.73 |
| Flavobacteriales_unclassified | 0.00 | 0.00 | 0.00 | 0.08 | 0.05 | 0.00 | 0.00 | 0.00 | 0.01 |
| Francisellaceae | 0.00 | 0.00 | 0.00 | 0.00 | 0.00 | 0.00 | 0.00 | 0.00 | 0.00 |
| Fusobacteriaceae | 0.19 | 0.38 | 0.02 | 0.01 | 0.00 | 0.01 | 0.02 | 0.01 | 0.01 |
| GIF3_norank | 0.00 | 0.00 | 0.00 | 0.00 | 0.00 | 0.00 | 0.00 | 0.00 | 0.00 |
| GIF9_norank | 0.00 | 0.00 | 0.00 | 0.00 | 0.00 | 0.00 | 0.00 | 0.00 | 0.00 |
| GR-WP33-30_norank | 0.00 | 0.01 | 0.00 | 0.00 | 0.00 | 0.00 | 0.00 | 0.00 | 0.00 |
| GR-WP33-58 | 0.00 | 0.00 | 0.00 | 0.00 | 0.00 | 0.00 | 0.00 | 0.00 | 0.00 |
| Gaiellaceae | 0.00 | 0.00 | 0.00 | 0.00 | 0.00 | 0.00 | 0.00 | 0.00 | 0.00 |
| Gaiellales_uncultured | 0.00 | 0.00 | 0.00 | 0.00 | 0.00 | 0.00 | 0.00 | 0.00 | 0.00 |
| Gammaproteobacteria_incertae_sedis | 0.21 | 0.60 | 0.19 | 0.24 | 0.09 | 0.10 | 0.02 | 0.04 | 0.03 |
| Gammaproteobacteria_unclassified | 0.05 | 0.13 | 0.03 | 0.15 | 0.09 | 0.11 | 0.01 | 0.00 | 0.01 |
| Gemmatimonadaceae | 0.00 | 0.00 | 0.00 | 0.00 | 0.00 | 0.00 | 0.00 | 0.00 | 0.00 |
| Geobacteraceae | 0.00 | 0.00 | 0.00 | 0.00 | 0.00 | 0.00 | 0.00 | 0.00 | 0.00 |
| Gracilibacteraceae | 0.00 | 0.00 | 0.00 | 0.00 | 0.00 | 0.00 | 0.00 | 0.00 | 0.00 |
| Granulosicoccaceae | 0.10 | 0.08 | 0.07 | 0.01 | 0.01 | 0.02 | 0.01 | 0.01 | 0.01 |

| | | | | | | | | | |
|---|---|---|---|---|---|---|---|---|---|
| HOC36_norank | 0.00 | 0.00 | 0.00 | 0.00 | 0.00 | 0.00 | 0.00 | 0.00 | 0.00 |
| Hahellaceae | 0.00 | 0.00 | 0.00 | 0.00 | 0.00 | 0.00 | 0.00 | 0.00 | 0.00 |
| Haliangiaceae | 0.00 | 0.00 | 0.00 | 0.00 | 0.00 | 0.00 | 0.00 | 0.00 | 0.00 |
| Halomonadaceae | 0.39 | 0.24 | 0.15 | 0.04 | 0.05 | 0.04 | 0.02 | 0.00 | 0.00 |
| Haloplasmataceae | 0.00 | 0.03 | 0.02 | 0.00 | 0.00 | 0.00 | 0.00 | 0.00 | 0.00 |
| Helicobacteraceae | 0.04 | 0.11 | 0.02 | 0.01 | 0.00 | 0.01 | 0.00 | 0.01 | 0.01 |
| Holosporaceae | 0.00 | 0.00 | 0.00 | 0.00 | 0.00 | 0.00 | 0.00 | 0.00 | 0.00 |
| Hydrogenophilaceae | 0.00 | 0.00 | 0.00 | 0.00 | 0.00 | 0.00 | 0.00 | 0.00 | 0.00 |
| Hyphomicrobiaceae | 0.08 | 0.18 | 0.04 | 0.01 | 0.01 | 0.02 | 0.01 | 0.01 | 0.01 |
| Hyphomonadaceae | 0.03 | 0.03 | 0.00 | 0.07 | 0.03 | 0.01 | 0.00 | 0.00 | 0.00 |
| Idiomarinaceae | 0.08 | 0.01 | 0.05 | 0.88 | 0.68 | 0.74 | 0.01 | 0.00 | 0.00 |
| Ignavibacteriaceae | 0.00 | 0.00 | 0.00 | 0.00 | 0.00 | 0.00 | 0.00 | 0.00 | 0.00 |
| Intrasporangiaceae | 0.00 | 0.00 | 0.00 | 0.00 | 0.00 | 0.00 | 0.00 | 0.00 | 0.00 |
| JG30-KF-CM45_norank | 0.00 | 0.00 | 0.00 | 0.00 | 0.00 | 0.00 | 0.00 | 0.00 | 0.00 |
| JG30-KF-CM66_norank | 0.00 | 0.00 | 0.00 | 0.00 | 0.00 | 0.00 | 0.00 | 0.00 | 0.00 |
| JTB215 | 0.00 | 0.06 | 0.00 | 0.00 | 0.00 | 0.00 | 0.03 | 0.05 | 0.00 |
| JTB255_marine_benthic_group | 0.23 | 0.30 | 0.14 | 0.05 | 0.06 | 0.08 | 0.03 | 0.06 | 0.02 |
| KI89A_clade_norank | 0.01 | 0.02 | 0.00 | 0.01 | 0.00 | 0.00 | 0.00 | 0.00 | 0.00 |
| Kazan-2B-17 | 0.04 | 0.01 | 0.00 | 0.00 | 0.00 | 0.00 | 0.00 | 0.00 | 0.00 |
| LD1-PA26_norank | 0.00 | 0.00 | 0.00 | 0.00 | 0.00 | 0.00 | 0.00 | 0.00 | 0.00 |
| LD1-PA34_norank | 0.00 | 0.01 | 0.00 | 0.00 | 0.00 | 0.00 | 0.00 | 0.00 | 0.00 |
| LD1-PB3_norank | 0.00 | 0.00 | 0.00 | 0.00 | 0.00 | 0.00 | 0.00 | 0.00 | 0.00 |

| | | | | | | | | | |
|---|---|---|---|---|---|---|---|---|---|
| LWSR-14 | 0.01 | 0.01 | 0.01 | 0.00 | 0.00 | 0.00 | 0.00 | 0.00 | 0.00 |
| Lachnospiraceae | 0.00 | 0.07 | 0.00 | 0.00 | 0.00 | 0.00 | 0.00 | 0.02 | 0.00 |
| Lactobacillaceae | 0.02 | 0.01 | 0.03 | 0.02 | 0.02 | 0.03 | 0.01 | 0.02 | 0.01 |
| Legionellaceae | 0.01 | 0.02 | 0.02 | 0.00 | 0.00 | 0.01 | 0.00 | 0.00 | 0.00 |
| Lentisphaeraceae | 0.00 | 0.00 | 0.00 | 0.00 | 0.00 | 0.00 | 0.00 | 0.00 | 0.00 |
| Lentisphaerae_unclassified | 0.00 | 0.00 | 0.00 | 0.00 | 0.00 | 0.00 | 0.00 | 0.00 | 0.00 |
| Leptospiraceae | 0.00 | 0.00 | 0.00 | 0.00 | 0.00 | 0.00 | 0.00 | 0.00 | 0.00 |
| Leptotrichiaceae | 0.00 | 0.00 | 0.01 | 0.00 | 0.01 | 0.00 | 0.00 | 0.00 | 0.00 |
| Leuconostocaceae | 0.11 | 0.03 | 0.22 | 0.04 | 0.07 | 0.10 | 0.06 | 0.12 | 0.06 |
| Lineage_I_(Endomicrobia)_norank | 0.00 | 0.00 | 0.00 | 0.00 | 0.00 | 0.00 | 0.00 | 0.00 | 0.00 |
| Listeriaceae | 0.08 | 0.03 | 0.13 | 0.10 | 0.09 | 0.07 | 0.06 | 0.04 | 0.05 |
| M2PB4-65_termite_group | 0.00 | 0.00 | 0.00 | 0.00 | 0.00 | 0.00 | 0.00 | 0.00 | 0.00 |
| MBAE14 | 0.00 | 0.00 | 0.00 | 0.03 | 0.00 | 0.00 | 0.00 | 0.00 | 0.00 |
| MSB-3A7_sediment_group_norank | 0.00 | 0.00 | 0.00 | 0.01 | 0.00 | 0.00 | 0.00 | 0.00 | 0.00 |
| Marinilabiaceae | 0.26 | 1.06 | 0.32 | 0.03 | 0.04 | 0.07 | 0.23 | 0.73 | 0.18 |
| Methylobacteriaceae | 0.00 | 0.00 | 0.00 | 0.00 | 0.00 | 0.00 | 0.00 | 0.00 | 0.00 |
| Methylococcaceae | 0.00 | 0.00 | 0.00 | 0.00 | 0.00 | 0.00 | 0.00 | 0.00 | 0.00 |
| Methylophilaceae | 0.26 | 0.21 | 0.16 | 0.09 | 0.13 | 0.09 | 0.08 | 0.04 | 0.06 |
| Microbacteriaceae | 0.01 | 0.01 | 0.01 | 0.00 | 0.00 | 0.00 | 0.00 | 0.00 | 0.00 |
| Micrococcaceae | 0.16 | 0.07 | 0.23 | 0.14 | 0.13 | 0.19 | 0.11 | 0.12 | 0.13 |
| Milano-WF1B-44_norank | 0.00 | 0.00 | 0.00 | 0.00 | 0.00 | 0.00 | 0.00 | 0.00 | 0.00 |
| Mollicutes_unclassified | 0.00 | 0.01 | 0.00 | 0.00 | 0.00 | 0.00 | 0.00 | 0.01 | 0.01 |

| | | | | | | | | | |
|---|---|---|---|---|---|---|---|---|---|
| Moraxellaceae | 0.29 | 1.67 | 0.59 | 0.49 | 0.46 | 0.38 | 0.32 | 0.12 | 0.29 |
| Moritellaceae | 0.00 | 0.00 | 0.00 | 0.00 | 0.00 | 0.00 | 0.00 | 0.00 | 0.00 |
| Mycobacteriaceae | 0.01 | 0.00 | 0.00 | 0.00 | 0.00 | 0.00 | 0.00 | 0.00 | 0.00 |
| Mycoplasmataceae | 0.00 | 0.00 | 0.00 | 0.00 | 0.00 | 0.00 | 0.00 | 0.00 | 0.00 |
| Myxococcales_unclassified | 0.00 | 0.00 | 0.00 | 0.00 | 0.00 | 0.00 | 0.00 | 0.00 | 0.00 |
| Myxococcales_uncultured | 0.00 | 0.00 | 0.00 | 0.00 | 0.00 | 0.00 | 0.00 | 0.00 | 0.00 |
| NB-1d | 0.00 | 0.00 | 0.00 | 0.07 | 0.08 | 0.12 | 0.02 | 0.01 | 0.04 |
| NB1-n_norank | 0.00 | 0.03 | 0.00 | 0.00 | 0.00 | 0.00 | 0.03 | 0.10 | 0.05 |
| NKB5_norank | 0.00 | 0.00 | 0.00 | 0.00 | 0.00 | 0.00 | 0.01 | 0.00 | 0.00 |
| NS11-12_marine_group | 0.00 | 0.01 | 0.01 | 0.01 | 0.00 | 0.00 | 0.00 | 0.00 | 0.01 |
| NS72 | 0.00 | 0.00 | 0.00 | 0.00 | 0.00 | 0.00 | 0.00 | 0.00 | 0.00 |
| NS7_marine_group | 0.01 | 0.00 | 0.00 | 0.00 | 0.00 | 0.00 | 0.00 | 0.00 | 0.00 |
| NS9_marine_group | 0.00 | 0.02 | 0.01 | 0.02 | 0.00 | 0.01 | 0.00 | 0.00 | 0.00 |
| Nannocystaceae | 0.00 | 0.00 | 0.00 | 0.00 | 0.00 | 0.00 | 0.00 | 0.00 | 0.00 |
| Neisseriaceae | 0.01 | 0.01 | 0.00 | 0.00 | 0.00 | 0.00 | 0.01 | 0.00 | 0.00 |
| Nitrosomonadaceae | 0.00 | 0.00 | 0.00 | 0.00 | 0.00 | 0.00 | 0.00 | 0.00 | 0.00 |
| Nitrospinaceae | 0.00 | 0.01 | 0.00 | 0.00 | 0.00 | 0.00 | 0.00 | 0.00 | 0.00 |
| Nitrospira_norank | 0.00 | 0.00 | 0.00 | 0.00 | 0.00 | 0.00 | 0.00 | 0.00 | 0.00 |
| Nocardiaceae | 0.05 | 0.01 | 0.01 | 0.01 | 0.02 | 0.02 | 0.00 | 0.00 | 0.00 |
| Nocardioidaceae | 0.00 | 0.00 | 0.00 | 0.01 | 0.00 | 0.01 | 0.00 | 0.00 | 0.00 |
| OCS116_clade_norank | 0.07 | 0.05 | 0.03 | 0.01 | 0.01 | 0.01 | 0.01 | 0.00 | 0.01 |
| OM190_norank | 0.04 | 0.16 | 0.04 | 0.01 | 0.02 | 0.01 | 0.01 | 0.01 | 0.01 |

| | | | | | | | | | |
|---|---|---|---|---|---|---|---|---|---|
| OM1_clade | 0.05 | 0.09 | 0.02 | 0.00 | 0.00 | 0.00 | 0.01 | 0.01 | 0.00 |
| OPB56 | 0.03 | 0.05 | 0.02 | 0.03 | 0.02 | 0.01 | 0.01 | 0.01 | 0.02 |
| Oceanospirillaceae | 5.64 | 1.45 | 2.30 | 2.19 | 2.62 | 2.54 | 8.62 | 1.68 | 5.70 |
| Oligosphaerales_norank | 0.00 | 0.00 | 0.00 | 0.00 | 0.00 | 0.00 | 0.00 | 0.00 | 0.00 |
| Opitutae_unclassified | 0.00 | 0.00 | 0.00 | 0.00 | 0.00 | 0.00 | 0.00 | 0.00 | 0.00 |
| Oxalobacteraceae | 0.01 | 0.00 | 0.05 | 0.01 | 0.03 | 0.02 | 0.00 | 0.02 | 0.00 |
| PAUC26f | 0.00 | 0.00 | 0.00 | 0.00 | 0.00 | 0.00 | 0.00 | 0.00 | 0.00 |
| PAUC43f_marine_benthic_group | 0.00 | 0.02 | 0.00 | 0.00 | 0.00 | 0.00 | 0.00 | 0.00 | 0.00 |
| PHOS-HE36 | 0.00 | 0.00 | 0.00 | 0.00 | 0.00 | 0.00 | 0.00 | 0.00 | 0.00 |
| PHOS-HE51 | 0.00 | 0.00 | 0.00 | 0.00 | 0.00 | 0.00 | 0.00 | 0.00 | 0.00 |
| Paenibacillaceae | 0.00 | 0.00 | 0.00 | 0.00 | 0.00 | 0.00 | 0.00 | 0.00 | 0.00 |
| Parvularculaceae | 0.02 | 0.01 | 0.01 | 0.01 | 0.00 | 0.00 | 0.00 | 0.00 | 0.01 |
| PeM15_norank | 0.01 | 0.02 | 0.02 | 0.00 | 0.00 | 0.01 | 0.00 | 0.00 | 0.00 |
| Peptostreptococcaceae | 0.20 | 0.21 | 0.08 | 0.00 | 0.00 | 0.01 | 0.03 | 0.37 | 0.00 |
| Phycisphaeraceae | 0.00 | 0.03 | 0.01 | 0.01 | 0.01 | 0.01 | 0.01 | 0.00 | 0.00 |
| Phyllobacteriaceae | 0.29 | 0.42 | 0.16 | 0.06 | 0.06 | 0.07 | 0.21 | 0.10 | 0.10 |
| Piscirickettsiaceae | 0.07 | 0.04 | 0.12 | 0.35 | 0.49 | 0.40 | 0.10 | 0.01 | 0.37 |
| Planctomycetaceae | 1.30 | 3.40 | 1.25 | 0.36 | 0.21 | 0.29 | 0.59 | 0.50 | 0.29 |
| Planctomycetes_unclassified | 0.00 | 0.00 | 0.00 | 0.00 | 0.00 | 0.00 | 0.00 | 0.00 | 0.00 |
| Planococcaceae | 0.06 | 0.05 | 0.11 | 0.04 | 0.09 | 0.10 | 0.09 | 0.05 | 0.05 |
| Porphyromonadaceae | 0.00 | 0.01 | 0.00 | 0.00 | 0.00 | 0.00 | 0.01 | 0.00 | 0.00 |
| Prevotellaceae | 0.00 | 0.00 | 0.00 | 0.00 | 0.00 | 0.00 | 0.00 | 0.00 | 0.00 |

| | | | | | | | | | |
|---|---|---|---|---|---|---|---|---|---|
| Propionibacteriaceae | 0.00 | 0.00 | 0.00 | 0.00 | 0.00 | 0.00 | 0.00 | 0.00 | 0.00 |
| Proteobacteria_unclassified | 0.00 | 0.00 | 0.00 | 0.00 | 0.00 | 0.00 | 0.00 | 0.00 | 0.00 |
| Pseudoalteromonadaceae | 21.83 | 8.40 | 22.93 | 20.16 | 24.99 | 15.93 | 9.91 | 6.95 | 13.28 |
| Pseudomonadaceae | 0.60 | 0.23 | 1.03 | 0.64 | 0.67 | 0.53 | 0.72 | 0.40 | 0.45 |
| Pseudonocardiaceae | 0.01 | 0.00 | 0.00 | 0.00 | 0.01 | 0.00 | 0.00 | 0.00 | 0.00 |
| Psychromonadaceae | 0.00 | 0.02 | 0.03 | 0.01 | 0.00 | 0.04 | 0.01 | 0.01 | 0.00 |
| Puniceicoccaceae | 0.01 | 0.01 | 0.00 | 0.00 | 0.00 | 0.00 | 0.00 | 0.00 | 0.00 |
| R76-B128_norank | 0.00 | 0.00 | 0.00 | 0.00 | 0.00 | 0.00 | 0.00 | 0.00 | 0.00 |
| RB41 | 0.00 | 0.00 | 0.00 | 0.00 | 0.00 | 0.00 | 0.00 | 0.00 | 0.00 |
| RF9_norank | 0.00 | 0.00 | 0.00 | 0.00 | 0.00 | 0.00 | 0.00 | 0.03 | 0.00 |
| Rhizobiaceae | 0.00 | 0.00 | 0.00 | 0.00 | 0.00 | 0.00 | 0.00 | 0.00 | 0.00 |
| Rhizobiales_unclassified | 0.00 | 0.01 | 0.00 | 0.00 | 0.00 | 0.00 | 0.00 | 0.00 | 0.00 |
| Rhizobiales_uncultured | 0.00 | 0.00 | 0.00 | 0.00 | 0.00 | 0.00 | 0.00 | 0.00 | 0.00 |
| Rhodobacteraceae | 5.35 | 10.34 | 5.65 | 3.39 | 2.75 | 3.18 | 1.01 | 0.74 | 0.92 |
| Rhodobiaceae | 0.06 | 0.09 | 0.04 | 0.03 | 0.00 | 0.02 | 0.00 | 0.00 | 0.02 |
| Rhodocyclaceae | 0.00 | 0.00 | 0.00 | 0.00 | 0.00 | 0.00 | 0.00 | 0.00 | 0.00 |
| Rhodospirillaceae | 0.01 | 0.01 | 0.01 | 0.01 | 0.00 | 0.00 | 0.00 | 0.00 | 0.03 |
| Rhodospirillales_rhodospirillales_incertae_sedis | 0.00 | 0.00 | 0.00 | 0.00 | 0.00 | 0.00 | 0.00 | 0.00 | 0.00 |
| Rhodospirillales_unclassified | 0.00 | 0.00 | 0.00 | 0.00 | 0.00 | 0.00 | 0.00 | 0.00 | 0.00 |
| Rhodothermaceae | 0.03 | 0.04 | 0.03 | 0.08 | 0.04 | 0.04 | 0.00 | 0.01 | 0.00 |
| Rickettsiaceae | 0.01 | 0.01 | 0.00 | 0.00 | 0.01 | 0.00 | 0.00 | 0.00 | 0.00 |
| Rickettsiales_rickettsiales_incertae_sedis | 0.00 | 0.00 | 0.00 | 0.00 | 0.00 | 0.00 | 0.00 | 0.00 | 0.00 |

| | | | | | | | | | |
|---|---|---|---|---|---|---|---|---|---|
| Rickettsiales_unclassified | 0.00 | 0.00 | 0.00 | 0.00 | 0.00 | 0.00 | 0.00 | 0.00 | 0.00 |
| Rubritaleaceae | 0.00 | 0.00 | 0.00 | 0.00 | 0.00 | 0.00 | 0.00 | 0.00 | 0.00 |
| Ruminococcaceae | 0.00 | 0.00 | 0.01 | 0.00 | 0.00 | 0.00 | 0.00 | 0.00 | 0.00 |
| Run-SP154_norank | 0.00 | 0.00 | 0.00 | 0.00 | 0.00 | 0.00 | 0.00 | 0.00 | 0.00 |
| SAR324_clade(Marine_group_B)_norank | 0.01 | 0.00 | 0.00 | 0.00 | 0.00 | 0.00 | 0.00 | 0.00 | 0.00 |
| SB-1_norank | 0.02 | 0.42 | 0.06 | 0.02 | 0.00 | 0.01 | 0.00 | 0.10 | 0.00 |
| SB-5_norank | 0.00 | 0.00 | 0.00 | 0.00 | 0.00 | 0.00 | 0.00 | 0.00 | 0.00 |
| SB1-18_norank | 0.00 | 0.00 | 0.00 | 0.01 | 0.00 | 0.00 | 0.00 | 0.00 | 0.00 |
| SC3-20_norank | 0.01 | 0.01 | 0.00 | 0.01 | 0.00 | 0.00 | 0.00 | 0.00 | 0.01 |
| SHA-109_norank | 0.02 | 0.01 | 0.01 | 0.00 | 0.00 | 0.00 | 0.02 | 0.00 | 0.01 |
| SJA-28 | 0.00 | 0.00 | 0.00 | 0.00 | 0.00 | 0.00 | 0.00 | 0.00 | 0.00 |
| SM2D12 | 0.00 | 0.00 | 0.00 | 0.00 | 0.00 | 0.00 | 0.00 | 0.00 | 0.00 |
| SPOTSOCT00m83_norank | 0.00 | 0.00 | 0.00 | 0.00 | 0.00 | 0.00 | 0.00 | 0.00 | 0.00 |
| SS1-B-03-39_norank | 0.00 | 0.01 | 0.00 | 0.00 | 0.00 | 0.00 | 0.00 | 0.00 | 0.00 |
| SS1-B-06-26 | 0.01 | 0.03 | 0.01 | 0.01 | 0.00 | 0.01 | 0.01 | 0.00 | 0.00 |
| SWB04 | 0.01 | 0.02 | 0.01 | 0.01 | 0.00 | 0.01 | 0.00 | 0.00 | 0.00 |
| SZB30_norank | 0.00 | 0.00 | 0.00 | 0.00 | 0.00 | 0.00 | 0.00 | 0.00 | 0.00 |
| Sandaracinaceae | 0.02 | 0.04 | 0.00 | 0.01 | 0.01 | 0.01 | 0.00 | 0.00 | 0.00 |
| Saprospiraceae | 0.17 | 0.28 | 0.13 | 0.10 | 0.06 | 0.12 | 0.03 | 0.06 | 0.01 |
| Schleiferiaceae | 0.00 | 0.00 | 0.00 | 0.00 | 0.00 | 0.00 | 0.00 | 0.00 | 0.00 |
| Sh765B-TzT-29_norank | 0.02 | 0.03 | 0.02 | 0.01 | 0.00 | 0.01 | 0.00 | 0.01 | 0.00 |
| Shewanellaceae | 0.31 | 0.29 | 0.10 | 0.68 | 0.91 | 1.59 | 0.07 | 0.03 | 0.04 |

| | | | | | | | | | |
|---|---|---|---|---|---|---|---|---|---|
| Skagenf62_norank | 0.00 | 0.00 | 0.00 | 0.00 | 0.00 | 0.00 | 0.01 | 0.00 | 0.01 |
| Solirubrobacteraceae | 0.00 | 0.00 | 0.00 | 0.00 | 0.00 | 0.00 | 0.00 | 0.00 | 0.00 |
| Sphingobacteriaceae | 0.00 | 0.01 | 0.01 | 0.01 | 0.01 | 0.00 | 0.00 | 0.00 | 0.01 |
| Sphingobacteriales_unclassified | 0.00 | 0.00 | 0.00 | 0.00 | 0.00 | 0.00 | 0.00 | 0.00 | 0.00 |
| Sphingomonadaceae | 0.02 | 0.04 | 0.03 | 0.02 | 0.03 | 0.01 | 0.01 | 0.01 | 0.01 |
| Sphingomonadales_unclassified | 0.01 | 0.01 | 0.01 | 0.01 | 0.01 | 0.00 | 0.02 | 0.00 | 0.00 |
| Spirochaetaceae | 0.00 | 0.00 | 0.00 | 0.00 | 0.00 | 0.00 | 0.00 | 0.00 | 0.00 |
| Staphylococcaceae | 0.00 | 0.01 | 0.01 | 0.00 | 0.00 | 0.00 | 0.01 | 0.00 | 0.01 |
| Streptococcaceae | 3.28 | 1.24 | 6.84 | 3.61 | 3.83 | 3.77 | 3.14 | 2.90 | 2.54 |
| Streptomycetaceae | 0.01 | 0.00 | 0.00 | 0.00 | 0.00 | 0.00 | 0.00 | 0.00 | 0.00 |
| Subgroup_17_norank | 0.00 | 0.01 | 0.00 | 0.00 | 0.00 | 0.00 | 0.00 | 0.00 | 0.00 |
| Subgroup_21_norank | 0.00 | 0.00 | 0.00 | 0.00 | 0.00 | 0.00 | 0.00 | 0.00 | 0.00 |
| Subgroup_23_norank | 0.01 | 0.03 | 0.00 | 0.00 | 0.00 | 0.01 | 0.00 | 0.00 | 0.00 |
| Subgroup_6_norank | 0.00 | 0.00 | 0.00 | 0.00 | 0.00 | 0.00 | 0.00 | 0.00 | 0.00 |
| Subgroup_9_norank | 0.00 | 0.01 | 0.01 | 0.00 | 0.00 | 0.00 | 0.00 | 0.00 | 0.00 |
| SubsectionIII_familyi | 0.01 | 0.03 | 0.00 | 0.00 | 0.00 | 0.00 | 0.00 | 0.00 | 0.00 |
| SubsectionII_familyii | 0.02 | 0.02 | 0.02 | 0.00 | 0.00 | 0.00 | 0.01 | 0.00 | 0.00 |
| SubsectionIV_familyii | 0.00 | 0.00 | 0.00 | 0.00 | 0.00 | 0.00 | 0.00 | 0.00 | 0.00 |
| SubsectionI_familyi | 0.04 | 0.07 | 0.03 | 0.01 | 0.00 | 0.00 | 0.01 | 0.01 | 0.00 |
| Surface_1 | 0.00 | 0.00 | 0.00 | 0.00 | 0.00 | 0.00 | 0.00 | 0.00 | 0.00 |
| Sva0071_norank | 0.03 | 0.05 | 0.01 | 0.01 | 0.00 | 0.00 | 0.01 | 0.00 | 0.00 |
| Sva0485_norank | 0.00 | 0.00 | 0.00 | 0.00 | 0.00 | 0.00 | 0.00 | 0.00 | 0.00 |

| | | | | | | | | | |
|---|---|---|---|---|---|---|---|---|---|
| Sva0725 | 0.02 | 0.06 | 0.01 | 0.00 | 0.00 | 0.00 | 0.00 | 0.01 | 0.01 |
| Sva0996_marine_group | 0.17 | 0.25 | 0.05 | 0.04 | 0.01 | 0.02 | 0.01 | 0.01 | 0.01 |
| Sva1033 | 0.00 | 0.01 | 0.00 | 0.00 | 0.00 | 0.00 | 0.00 | 0.00 | 0.00 |
| Synergistaceae | 0.00 | 0.00 | 0.00 | 0.00 | 0.00 | 0.00 | 0.00 | 0.00 | 0.00 |
| Syntrophaceae | 0.00 | 0.00 | 0.00 | 0.00 | 0.00 | 0.00 | 0.00 | 0.00 | 0.00 |
| Syntrophobacteraceae | 0.00 | 0.01 | 0.00 | 0.01 | 0.00 | 0.00 | 0.00 | 0.00 | 0.00 |
| Syntrophomonadaceae | 0.00 | 0.00 | 0.00 | 0.00 | 0.00 | 0.00 | 0.00 | 0.00 | 0.00 |
| Syntrophorhabdaceae | 0.00 | 0.00 | 0.00 | 0.00 | 0.00 | 0.00 | 0.00 | 0.00 | 0.00 |
| TA18_norank | 0.03 | 0.01 | 0.00 | 0.00 | 0.00 | 0.00 | 0.00 | 0.01 | 0.01 |
| TK34 | 0.00 | 0.00 | 0.00 | 0.00 | 0.00 | 0.00 | 0.00 | 0.00 | 0.00 |
| TK85 | 0.02 | 0.02 | 0.01 | 0.00 | 0.00 | 0.00 | 0.00 | 0.00 | 0.00 |
| TM6_norank | 0.00 | 0.00 | 0.00 | 0.00 | 0.00 | 0.00 | 0.00 | 0.00 | 0.00 |
| Thermoanaerobacteraceae | 0.00 | 0.00 | 0.00 | 0.00 | 0.00 | 0.00 | 0.00 | 0.00 | 0.00 |
| Thiotrichaceae | 0.30 | 0.26 | 0.11 | 0.05 | 0.02 | 0.05 | 0.09 | 0.06 | 0.07 |
| Thiotrichales_thiotrichales_incertae_sedis | 0.00 | 0.00 | 0.00 | 0.00 | 0.00 | 0.00 | 0.00 | 0.00 | 0.00 |
| Trueperaceae | 0.00 | 0.01 | 0.00 | 0.00 | 0.00 | 0.00 | 0.00 | 0.00 | 0.00 |
| Unknown_Family | 0.05 | 0.09 | 0.04 | 0.02 | 0.03 | 0.04 | 0.01 | 0.47 | 0.01 |
| VC2.1_Bac22_norank | 0.00 | 0.01 | 0.00 | 0.00 | 0.00 | 0.00 | 0.00 | 0.01 | 0.00 |
| VHS-B3-70 | 0.00 | 0.00 | 0.00 | 0.00 | 0.00 | 0.00 | 0.00 | 0.00 | 0.00 |
| Veillonellaceae | 0.00 | 0.00 | 0.00 | 0.00 | 0.00 | 0.00 | 0.00 | 0.00 | 0.00 |
| Verrucomicrobiaceae | 0.03 | 0.01 | 0.01 | 0.01 | 0.00 | 0.00 | 0.01 | 0.01 | 0.00 |
| Vibrionaceae | 19.06 | 19.03 | 30.22 | 10.49 | 10.58 | 19.96 | 36.99 | 45.40 | 35.61 |

| | | | | | | | | | |
|---|---|---|---|---|---|---|---|---|---|
| WCHB1-69 | 0.00 | 0.00 | 0.00 | 0.00 | 0.00 | 0.00 | 0.00 | 0.00 | 0.00 |
| WD2101_soil_group_norank | 0.00 | 0.00 | 0.00 | 0.00 | 0.00 | 0.00 | 0.00 | 0.00 | 0.00 |
| Xanthobacteraceae | 0.00 | 0.00 | 0.00 | 0.00 | 0.00 | 0.00 | 0.00 | 0.00 | 0.00 |
| Xanthomonadaceae | 0.01 | 0.01 | 0.02 | 0.01 | 0.01 | 0.00 | 0.01 | 0.00 | 0.01 |
| Xanthomonadales_unclassified | 0.00 | 0.00 | 0.00 | 0.01 | 0.01 | 0.00 | 0.00 | 0.00 | 0.00 |
| Xanthomonadales_uncultured | 0.00 | 0.01 | 0.00 | 0.00 | 0.00 | 0.00 | 0.00 | 0.00 | 0.00 |
| Xanthomonadales_xanthomonadales_incertae_sedis | 0.00 | 0.00 | 0.00 | 0.00 | 0.00 | 0.00 | 0.00 | 0.00 | 0.00 |
| mitochondria | 0.00 | 0.00 | 0.00 | 0.00 | 0.00 | 0.00 | 0.00 | 0.00 | 0.00 |
| ratAN060301C | 0.00 | 0.00 | 0.01 | 0.00 | 0.01 | 0.00 | 0.00 | 0.00 | 0.00 |
| ss1-B-07-44_norank | 0.00 | 0.00 | 0.00 | 0.00 | 0.00 | 0.00 | 0.00 | 0.00 | 0.00 |
| vadinBA26_norank | 0.00 | 0.00 | 0.00 | 0.00 | 0.00 | 0.00 | 0.00 | 0.00 | 0.00 |
| vadinHA17_norank | 0.00 | 0.00 | 0.00 | 0.00 | 0.00 | 0.00 | 0.00 | 0.00 | 0.00 |

**Table S3.** Relative abundance (in %) of the sequences from biofilms on glass, PDMS and PDMS filled with 1 wt% AgNPs assigned to different genera.

| **Genus** | **Glass-1** | **Glass-2** | **Glass-3** | **PDMS-1** | **PDMS-2** | **PDMS-3** | **1 wt% AgNPs-1** | **1 wt% AgNPs-2** | **1 wt% AgNPs-3** |
|---|---|---|---|---|---|---|---|---|---|
| 0319-6G20_norank | 0.00 | 0.00 | 0.00 | 0.00 | 0.00 | 0.00 | 0.00 | 0.00 | 0.00 |
| 288-2_norank | 0.00 | 0.00 | 0.00 | 0.00 | 0.00 | 0.00 | 0.00 | 0.00 | 0.00 |
| 34P16_norank | 0.00 | 0.00 | 0.00 | 0.00 | 0.00 | 0.00 | 0.00 | 0.00 | 0.00 |
| 43F-1404R_norank | 0.00 | 0.00 | 0.00 | 0.00 | 0.00 | 0.00 | 0.00 | 0.00 | 0.00 |
| ABS-19_norank | 0.00 | 0.00 | 0.00 | 0.00 | 0.00 | 0.00 | 0.00 | 0.00 | 0.00 |
| AEGEAN-169_marine_group | 0.00 | 0.00 | 0.00 | 0.00 | 0.00 | 0.00 | 0.00 | 0.00 | 0.00 |
| AEGEAN-245_norank | 0.00 | 0.00 | 0.00 | 0.00 | 0.00 | 0.00 | 0.00 | 0.00 | 0.00 |
| AKIW1012_norank | 0.00 | 0.00 | 0.00 | 0.00 | 0.00 | 0.00 | 0.00 | 0.00 | 0.00 |
| AT-s3-28_norank | 0.00 | 0.00 | 0.00 | 0.00 | 0.00 | 0.00 | 0.00 | 0.00 | 0.00 |
| Acetobacter | 0.00 | 0.00 | 0.00 | 0.00 | 0.00 | 0.00 | 0.00 | 0.00 | 0.00 |
| Acholeplasma | 0.07 | 0.01 | 0.00 | 0.00 | 0.00 | 0.00 | 0.02 | 0.04 | 0.07 |
| Achromobacter | 0.00 | 0.00 | 0.00 | 0.00 | 0.00 | 0.00 | 0.00 | 0.00 | 0.00 |
| Acidaminococcaceae_uncultured | 0.00 | 0.00 | 0.00 | 0.00 | 0.00 | 0.00 | 0.00 | 0.00 | 0.00 |
| Acidiferrobacter | 0.01 | 0.03 | 0.01 | 0.01 | 0.00 | 0.01 | 0.00 | 0.00 | 0.00 |
| Acidimicrobiaceae_uncultured | 0.10 | 0.16 | 0.04 | 0.02 | 0.00 | 0.00 | 0.01 | 0.01 | 0.00 |
| Acidimicrobiales_unclassified | 0.00 | 0.00 | 0.00 | 0.00 | 0.00 | 0.00 | 0.00 | 0.00 | 0.00 |
| Acidimicrobiales_uncultured | 0.07 | 0.11 | 0.02 | 0.01 | 0.00 | 0.00 | 0.02 | 0.01 | 0.00 |
| Acidobacteria_norank | 0.01 | 0.01 | 0.00 | 0.00 | 0.00 | 0.00 | 0.00 | 0.00 | 0.00 |

| | | | | | | | | | |
|---|---|---|---|---|---|---|---|---|---|
| Acinetobacter | 0.05 | 0.02 | 0.10 | 0.05 | 0.02 | 0.01 | 0.04 | 0.02 | 0.01 |
| Actibacter | 0.08 | 0.48 | 0.17 | 0.11 | 0.02 | 0.08 | 0.03 | 0.05 | 0.01 |
| Actinobacteria_norank | 0.00 | 0.00 | 0.00 | 0.00 | 0.00 | 0.00 | 0.00 | 0.00 | 0.00 |
| Adriatic90_norank | 0.00 | 0.00 | 0.00 | 0.00 | 0.00 | 0.00 | 0.00 | 0.00 | 0.00 |
| Advenella | 0.00 | 0.00 | 0.00 | 0.00 | 0.00 | 0.00 | 0.00 | 0.00 | 0.00 |
| Aequorivita | 0.03 | 0.01 | 0.02 | 0.01 | 0.02 | 0.02 | 0.00 | 0.00 | 0.00 |
| Aerococcaceae_uncultured | 0.00 | 0.00 | 0.01 | 0.00 | 0.00 | 0.00 | 0.00 | 0.00 | 0.00 |
| Aerococcus | 0.00 | 0.00 | 0.00 | 0.00 | 0.00 | 0.00 | 0.00 | 0.00 | 0.00 |
| Aeromonas | 0.00 | 0.00 | 0.00 | 0.00 | 0.00 | 0.00 | 0.00 | 0.00 | 0.00 |
| Afifella | 0.01 | 0.00 | 0.01 | 0.00 | 0.00 | 0.00 | 0.00 | 0.00 | 0.00 |
| Agarivorans | 0.01 | 0.00 | 0.00 | 0.00 | 0.00 | 0.01 | 0.00 | 0.00 | 0.00 |
| Albimonas | 0.02 | 0.01 | 0.00 | 0.01 | 0.00 | 0.00 | 0.00 | 0.00 | 0.00 |
| Alcaligenaceae_uncultured | 0.00 | 0.00 | 0.00 | 0.00 | 0.00 | 0.00 | 0.00 | 0.00 | 0.00 |
| Alcaligenes | 0.00 | 0.00 | 0.00 | 0.00 | 0.00 | 0.00 | 0.00 | 0.00 | 0.00 |
| Alcanivoracaceae_norank | 0.08 | 0.01 | 0.05 | 1.16 | 1.36 | 0.68 | 0.00 | 0.00 | 0.05 |
| Algibacter | 0.70 | 0.72 | 0.38 | 1.10 | 0.79 | 1.11 | 0.29 | 0.27 | 0.51 |
| Algimonas | 0.00 | 0.00 | 0.00 | 0.01 | 0.01 | 0.00 | 0.00 | 0.00 | 0.00 |
| Algoriphagus | 0.00 | 0.00 | 0.00 | 0.00 | 0.00 | 0.00 | 0.00 | 0.00 | 0.00 |
| Aliidiomarina | 0.00 | 0.00 | 0.00 | 0.03 | 0.01 | 0.03 | 0.00 | 0.00 | 0.00 |
| Alkaliphilus | 0.01 | 0.00 | 0.00 | 0.00 | 0.00 | 0.00 | 0.00 | 0.00 | 0.00 |
| Alphaproteobacteria_unclassified | 0.02 | 0.01 | 0.00 | 0.00 | 0.00 | 0.01 | 0.00 | 0.00 | 0.00 |
| Altererythrobacter | 0.03 | 0.02 | 0.02 | 0.04 | 0.01 | 0.00 | 0.00 | 0.00 | 0.00 |

| | | | | | | | | | |
|---|---|---|---|---|---|---|---|---|---|
| Alteromonadaceae_norank | 0.00 | 0.01 | 0.00 | 0.00 | 0.00 | 0.00 | 0.00 | 0.00 | 0.00 |
| Alteromonadaceae_unclassified | 0.06 | 0.13 | 0.05 | 0.83 | 1.24 | 0.89 | 0.10 | 0.01 | 0.28 |
| Alteromonadales_unclassified | 0.01 | 0.01 | 0.03 | 0.00 | 0.00 | 0.00 | 0.00 | 0.00 | 0.00 |
| Alteromonas | 0.04 | 0.03 | 0.03 | 0.44 | 0.69 | 1.10 | 0.08 | 0.01 | 0.18 |
| Amaricoccus | 0.00 | 0.00 | 0.00 | 0.00 | 0.00 | 0.00 | 0.00 | 0.00 | 0.00 |
| Amphritea | 0.00 | 0.00 | 0.00 | 0.02 | 0.04 | 0.03 | 0.02 | 0.01 | 0.14 |
| Anaerolinea | 0.00 | 0.00 | 0.00 | 0.00 | 0.00 | 0.00 | 0.00 | 0.00 | 0.00 |
| Anaerolineaceae_unclassified | 0.00 | 0.00 | 0.00 | 0.00 | 0.00 | 0.00 | 0.00 | 0.00 | 0.00 |
| Anaerolineaceae_uncultured | 0.01 | 0.04 | 0.01 | 0.01 | 0.00 | 0.00 | 0.00 | 0.01 | 0.00 |
| Anaeromyxobacter | 0.00 | 0.00 | 0.00 | 0.00 | 0.00 | 0.00 | 0.00 | 0.00 | 0.00 |
| Anderseniella | 0.03 | 0.04 | 0.03 | 0.01 | 0.00 | 0.00 | 0.00 | 0.00 | 0.01 |
| Anoxybacillus | 0.01 | 0.00 | 0.00 | 0.00 | 0.00 | 0.00 | 0.00 | 0.00 | 0.00 |
| Aquicella | 0.01 | 0.02 | 0.02 | 0.00 | 0.00 | 0.01 | 0.01 | 0.00 | 0.01 |
| Aquimarina | 0.00 | 0.02 | 0.04 | 0.07 | 0.01 | 0.07 | 0.00 | 0.00 | 0.00 |
| Arcobacter | 6.72 | 1.97 | 0.71 | 0.04 | 0.07 | 0.16 | 3.75 | 10.16 | 3.90 |
| Ardenticatenales_norank | 0.00 | 0.00 | 0.00 | 0.00 | 0.00 | 0.00 | 0.00 | 0.00 | 0.00 |
| Ardenticatenia_uncultured | 0.00 | 0.04 | 0.01 | 0.00 | 0.00 | 0.00 | 0.00 | 0.00 | 0.00 |
| Arenibacter | 0.18 | 0.03 | 0.06 | 0.12 | 0.17 | 0.18 | 0.00 | 0.00 | 0.12 |
| Arenicella | 0.03 | 0.06 | 0.03 | 0.02 | 0.03 | 0.03 | 0.01 | 0.00 | 0.01 |
| Arenimonas | 0.00 | 0.00 | 0.00 | 0.00 | 0.00 | 0.00 | 0.00 | 0.00 | 0.00 |
| Armatimonadetes_norank | 0.00 | 0.00 | 0.00 | 0.00 | 0.00 | 0.00 | 0.00 | 0.00 | 0.00 |
| Arthrobacter | 0.16 | 0.07 | 0.23 | 0.14 | 0.13 | 0.19 | 0.11 | 0.12 | 0.13 |

| | | | | | | | | | |
|---|---|---|---|---|---|---|---|---|---|
| Arthrospira | 0.01 | 0.03 | 0.00 | 0.00 | 0.00 | 0.00 | 0.00 | 0.00 | 0.00 |
| Atopostipes | 0.00 | 0.00 | 0.00 | 0.00 | 0.00 | 0.00 | 0.01 | 0.00 | 0.00 |
| Aurantimonas | 0.00 | 0.00 | 0.00 | 0.00 | 0.00 | 0.00 | 0.00 | 0.00 | 0.00 |
| Aureicoccus | 0.00 | 0.01 | 0.00 | 0.00 | 0.00 | 0.00 | 0.00 | 0.00 | 0.00 |
| Aureispira | 0.00 | 0.00 | 0.00 | 0.00 | 0.00 | 0.00 | 0.00 | 0.00 | 0.00 |
| BD1-5_norank | 1.81 | 0.72 | 0.56 | 0.05 | 0.33 | 0.18 | 12.14 | 0.98 | 2.53 |
| BD1-7_clade | 0.00 | 0.00 | 0.00 | 0.02 | 0.00 | 0.00 | 0.00 | 0.00 | 0.00 |
| BD2-11_terrestrial_group_norank | 0.00 | 0.00 | 0.00 | 0.00 | 0.00 | 0.00 | 0.00 | 0.00 | 0.00 |
| BD2-2_norank | 0.00 | 0.01 | 0.00 | 0.01 | 0.00 | 0.00 | 0.00 | 0.00 | 0.00 |
| BD7-8_marine_group_norank | 0.00 | 0.00 | 0.00 | 0.00 | 0.00 | 0.00 | 0.00 | 0.00 | 0.00 |
| BG.g7_norank | 0.00 | 0.00 | 0.00 | 0.00 | 0.00 | 0.00 | 0.00 | 0.00 | 0.00 |
| BSV26_norank | 0.00 | 0.00 | 0.00 | 0.00 | 0.00 | 0.00 | 0.00 | 0.00 | 0.00 |
| Bacillus | 0.08 | 0.02 | 0.09 | 0.05 | 0.05 | 0.07 | 0.04 | 0.06 | 0.07 |
| Bacteria_unclassified | 0.03 | 0.85 | 0.05 | 0.02 | 0.01 | 0.49 | 0.36 | 0.64 | 0.07 |
| Bacteriovoracaceae_unclassified | 0.00 | 0.00 | 0.00 | 0.00 | 0.00 | 0.00 | 0.00 | 0.00 | 0.00 |
| Bacteriovoracaceae_uncultured | 0.00 | 0.00 | 0.00 | 0.00 | 0.00 | 0.00 | 0.00 | 0.00 | 0.00 |
| Bacteriovorax | 0.01 | 0.00 | 0.00 | 9.44 | 7.29 | 3.53 | 0.00 | 0.00 | 0.00 |
| Bacteroidales_unclassified | 0.00 | 0.02 | 0.00 | 0.00 | 0.00 | 0.00 | 0.01 | 0.01 | 0.00 |
| Bacteroides | 0.00 | 0.08 | 0.00 | 0.00 | 0.00 | 0.00 | 0.06 | 0.36 | 0.00 |
| Bacteroidetes_unclassified | 0.00 | 0.01 | 0.01 | 0.05 | 0.08 | 0.02 | 0.00 | 0.00 | 0.00 |
| Beutenbergia | 0.00 | 0.01 | 0.00 | 0.00 | 0.00 | 0.00 | 0.00 | 0.00 | 0.00 |

| | | | | | | | | | |
|---|---|---|---|---|---|---|---|---|---|
| Bifidobacterium | 0.00 | 0.01 | 0.02 | 0.00 | 0.00 | 0.01 | 0.00 | 0.00 | 0.00 |
| Bizionia | 2.63 | 0.20 | 0.36 | 1.29 | 1.64 | 2.22 | 0.15 | 0.01 | 0.02 |
| Blastocatella | 0.02 | 0.01 | 0.00 | 0.00 | 0.00 | 0.01 | 0.00 | 0.00 | 0.00 |
| Blastopirellula | 0.44 | 1.23 | 0.47 | 0.13 | 0.06 | 0.11 | 0.21 | 0.15 | 0.09 |
| Brevibacterium | 0.00 | 0.00 | 0.00 | 0.00 | 0.00 | 0.00 | 0.00 | 0.00 | 0.00 |
| Brevundimonas | 0.00 | 0.00 | 0.00 | 0.00 | 0.00 | 0.00 | 0.00 | 0.00 | 0.00 |
| Brochothrix | 0.08 | 0.03 | 0.13 | 0.10 | 0.09 | 0.07 | 0.06 | 0.04 | 0.05 |
| Brumimicrobium | 0.00 | 0.01 | 0.04 | 0.59 | 0.71 | 0.28 | 0.00 | 0.05 | 0.08 |
| Burkholderiaceae_unclassified | 0.01 | 0.00 | 0.00 | 0.00 | 0.00 | 0.00 | 0.00 | 0.00 | 0.00 |
| Bythopirellula | 0.04 | 0.09 | 0.02 | 0.01 | 0.00 | 0.00 | 0.02 | 0.00 | 0.00 |
| C1-B045 | 0.00 | 0.00 | 0.00 | 0.00 | 0.00 | 0.00 | 0.00 | 0.00 | 0.00 |
| CA002_norank | 0.00 | 0.00 | 0.00 | 0.00 | 0.00 | 0.00 | 0.00 | 0.00 | 0.00 |
| CKC4_norank | 0.10 | 3.50 | 0.01 | 0.00 | 0.00 | 0.01 | 2.90 | 1.72 | 0.67 |
| Caenarcaniphilales_norank | 0.00 | 0.00 | 0.00 | 0.00 | 0.00 | 0.00 | 0.00 | 0.00 | 0.00 |
| Caldilineaceae_uncultured | 0.00 | 0.01 | 0.00 | 0.00 | 0.00 | 0.00 | 0.00 | 0.00 | 0.00 |
| Caldithrix | 0.00 | 0.00 | 0.00 | 0.00 | 0.00 | 0.00 | 0.00 | 0.00 | 0.00 |
| Candidate_division_BRC1_norank | 0.00 | 0.00 | 0.00 | 0.00 | 0.00 | 0.00 | 0.00 | 0.00 | 0.00 |
| Candidate_division_OD1_norank | 0.00 | 0.00 | 0.00 | 0.00 | 0.00 | 0.00 | 0.00 | 0.00 | 0.00 |
| Candidate_division_OP11_norank | 0.00 | 0.00 | 0.00 | 0.00 | 0.00 | 0.00 | 0.00 | 0.00 | 0.00 |
| Candidate_division_OP3_norank | 0.00 | 0.00 | 0.00 | 0.00 | 0.00 | 0.00 | 0.00 | 0.00 | 0.00 |
| Candidate_division_OP8_norank | 0.00 | 0.00 | 0.00 | 0.00 | 0.00 | 0.00 | 0.00 | 0.00 | 0.00 |
| Candidate_division_SR1_norank | 0.00 | 0.00 | 0.00 | 0.00 | 0.00 | 0.00 | 0.00 | 0.00 | 0.00 |

| | | | | | | | | | |
|---|---|---|---|---|---|---|---|---|---|
| Candidate_division_TM7_norank | 0.03 | 0.06 | 0.01 | 0.00 | 0.00 | 0.01 | 0.00 | 0.00 | 0.01 |
| Candidate_division_TM7_unclassified | 0.00 | 0.00 | 0.00 | 0.00 | 0.00 | 0.00 | 0.00 | 0.00 | 0.00 |
| Candidate_division_WS3_norank | 0.01 | 0.01 | 0.00 | 0.01 | 0.00 | 0.00 | 0.00 | 0.00 | 0.00 |
| Candidatus_Amoebophilus | 0.00 | 0.00 | 0.00 | 0.00 | 0.00 | 0.00 | 0.00 | 0.00 | 0.00 |
| Candidatus_Competibacter | 0.00 | 0.00 | 0.00 | 0.00 | 0.00 | 0.00 | 0.00 | 0.00 | 0.00 |
| Candidatus_Endobugula | 0.00 | 0.00 | 0.00 | 0.00 | 0.00 | 0.00 | 0.00 | 0.00 | 0.00 |
| Candidatus_Endoecteinascidia | 0.00 | 0.00 | 0.00 | 0.00 | 0.00 | 0.00 | 0.00 | 0.00 | 0.00 |
| Candidatus_Lariskella | 0.00 | 0.00 | 0.00 | 0.00 | 0.00 | 0.00 | 0.00 | 0.00 | 0.00 |
| Candidatus_Thiobios | 0.00 | 0.00 | 0.00 | 0.00 | 0.00 | 0.00 | 0.00 | 0.00 | 0.00 |
| Carnobacteriaceae_unclassified | 0.04 | 0.00 | 0.02 | 0.00 | 0.00 | 0.00 | 0.01 | 0.00 | 0.00 |
| Carnobacterium | 0.03 | 0.01 | 0.05 | 0.01 | 0.02 | 0.03 | 0.02 | 0.01 | 0.03 |
| Castellaniella | 0.00 | 0.00 | 0.00 | 0.00 | 0.00 | 0.00 | 0.00 | 0.00 | 0.00 |
| Catenovulum | 0.00 | 0.00 | 0.00 | 0.00 | 0.00 | 0.03 | 0.00 | 0.00 | 0.00 |
| Cellulophaga | 0.00 | 0.00 | 0.00 | 0.00 | 0.00 | 0.00 | 0.00 | 0.00 | 0.00 |
| Cerasicoccus | 0.00 | 0.00 | 0.00 | 0.00 | 0.00 | 0.00 | 0.00 | 0.00 | 0.00 |
| Cetobacterium | 0.00 | 0.00 | 0.00 | 0.00 | 0.00 | 0.00 | 0.00 | 0.00 | 0.00 |
| Chitinophagaceae_unclassified | 0.00 | 0.01 | 0.00 | 0.00 | 0.00 | 0.00 | 0.00 | 0.00 | 0.00 |
| Chitinophagaceae_uncultured | 0.00 | 0.01 | 0.00 | 0.00 | 0.01 | 0.00 | 0.00 | 0.00 | 0.00 |
| Chlamydiaceae_uncultured | 0.00 | 0.00 | 0.00 | 0.00 | 0.00 | 0.00 | 0.00 | 0.00 | 0.00 |
| Chlamydiales_unclassified | 0.00 | 0.00 | 0.00 | 0.00 | 0.00 | 0.00 | 0.00 | 0.00 | 0.00 |
| Chloroflexi_uncultured | 0.00 | 0.00 | 0.00 | 0.00 | 0.00 | 0.00 | 0.00 | 0.00 | 0.00 |
| Chromatiaceae_uncultured | 0.00 | 0.00 | 0.00 | 0.00 | 0.00 | 0.00 | 0.00 | 0.00 | 0.00 |

| | | | | | | | | | |
|---|---|---|---|---|---|---|---|---|---|
| Chryseobacterium | 0.03 | 0.00 | 0.01 | 0.01 | 0.01 | 0.01 | 0.02 | 0.01 | 0.01 |
| Clostridiaceae_1_unclassified | 0.01 | 0.01 | 0.00 | 0.00 | 0.00 | 0.00 | 0.00 | 0.00 | 0.00 |
| Clostridiales_unclassified | 0.02 | 0.05 | 0.01 | 0.00 | 0.00 | 0.00 | 0.11 | 0.02 | 0.02 |
| Clostridiisalibacter | 0.08 | 0.06 | 0.22 | 0.00 | 0.00 | 0.00 | 0.02 | 0.03 | 0.00 |
| Clostridium_sensu_stricto_1 | 0.01 | 0.01 | 0.00 | 0.00 | 0.00 | 0.00 | 0.00 | 0.00 | 0.00 |
| Clostridium_sensu_stricto_17 | 3.57 | 0.25 | 0.67 | 0.00 | 0.00 | 0.00 | 0.09 | 0.22 | 0.00 |
| Clostridium_sensu_stricto_18 | 0.04 | 0.01 | 0.02 | 0.00 | 0.00 | 0.00 | 0.00 | 0.00 | 0.00 |
| Clostridium_sensu_stricto_5 | 0.00 | 0.00 | 0.00 | 0.00 | 0.00 | 0.00 | 0.00 | 0.00 | 0.00 |
| Clostridium_sensu_stricto_7 | 0.00 | 0.01 | 0.00 | 0.00 | 0.00 | 0.00 | 0.00 | 0.00 | 0.00 |
| Cobetia | 0.00 | 0.00 | 0.05 | 0.00 | 0.01 | 0.01 | 0.00 | 0.00 | 0.00 |
| Cocleimonas | 0.01 | 0.01 | 0.01 | 0.00 | 0.00 | 0.00 | 0.00 | 0.00 | 0.00 |
| Cohaesibacter | 0.01 | 0.02 | 0.00 | 0.00 | 0.00 | 0.01 | 0.01 | 0.00 | 0.02 |
| Colwellia | 0.01 | 0.00 | 0.01 | 0.70 | 0.57 | 1.35 | 0.01 | 0.01 | 0.03 |
| Colwelliaceae_unclassified | 0.00 | 0.00 | 0.01 | 0.07 | 0.04 | 0.16 | 0.00 | 0.01 | 0.01 |
| Comamonadaceae_unclassified | 0.00 | 0.00 | 0.00 | 0.01 | 0.00 | 0.00 | 0.01 | 0.00 | 0.00 |
| Comamonas | 0.00 | 0.00 | 0.01 | 0.00 | 0.00 | 0.00 | 0.00 | 0.00 | 0.00 |
| Congregibacter | 0.00 | 0.00 | 0.01 | 0.03 | 0.02 | 0.02 | 0.00 | 0.00 | 0.00 |
| Corynebacterium | 0.00 | 0.00 | 0.01 | 0.00 | 0.00 | 0.00 | 0.00 | 0.01 | 0.00 |
| Coxiella | 0.00 | 0.01 | 0.00 | 0.00 | 0.00 | 0.00 | 0.00 | 0.00 | 0.00 |
| Coxiellaceae_uncultured | 0.00 | 0.01 | 0.00 | 0.00 | 0.00 | 0.00 | 0.00 | 0.00 | 0.00 |
| Crenothrix | 0.00 | 0.00 | 0.00 | 0.00 | 0.00 | 0.00 | 0.00 | 0.00 | 0.00 |
| Croceibacter | 0.00 | 0.00 | 0.00 | 0.00 | 0.00 | 0.00 | 0.00 | 0.00 | 0.00 |

| | | | | | | | | | |
|---|---|---|---|---|---|---|---|---|---|
| Crocinitomix | 0.01 | 0.01 | 0.01 | 0.04 | 0.00 | 0.00 | 0.00 | 0.00 | 0.00 |
| Cryomorpha | 0.00 | 0.00 | 0.01 | 0.01 | 0.00 | 0.01 | 0.00 | 0.00 | 0.00 |
| Cryomorphaceae_unclassified | 0.02 | 0.02 | 0.00 | 0.41 | 0.61 | 0.09 | 0.00 | 0.01 | 0.01 |
| Cryomorphaceae_uncultured | 0.00 | 0.02 | 0.00 | 0.02 | 0.00 | 0.00 | 0.00 | 0.00 | 0.00 |
| Cyanobacteria_norank | 0.06 | 0.08 | 0.04 | 0.01 | 0.02 | 0.03 | 0.01 | 0.01 | 0.00 |
| Cyclobacterium | 0.01 | 0.00 | 0.00 | 0.00 | 0.00 | 0.00 | 0.00 | 0.00 | 0.00 |
| Cycloclasticus | 0.00 | 0.00 | 0.00 | 0.00 | 0.00 | 0.00 | 0.00 | 0.00 | 0.00 |
| Cytophagaceae_unclassified | 0.00 | 0.00 | 0.00 | 0.00 | 0.00 | 0.00 | 0.00 | 0.00 | 0.00 |
| Cytophagaceae_uncultured | 0.00 | 0.00 | 0.00 | 0.00 | 0.00 | 0.00 | 0.00 | 0.00 | 0.00 |
| Cytophagales_unclassified | 0.00 | 0.01 | 0.00 | 0.00 | 0.00 | 0.00 | 0.00 | 0.00 | 0.00 |
| DB1-14_norank | 0.14 | 0.13 | 0.12 | 0.01 | 0.03 | 0.07 | 0.05 | 0.05 | 0.03 |
| DEV007_norank | 0.02 | 0.01 | 0.00 | 0.00 | 0.00 | 0.00 | 0.00 | 0.00 | 0.00 |
| Dasania | 0.00 | 0.00 | 0.00 | 0.00 | 0.00 | 0.00 | 0.00 | 0.00 | 0.00 |
| Defluviicoccus | 0.00 | 0.00 | 0.00 | 0.00 | 0.00 | 0.00 | 0.00 | 0.00 | 0.00 |
| Defluviitaleaceae_uncultured | 0.32 | 0.04 | 0.00 | 0.00 | 0.00 | 0.00 | 0.08 | 0.04 | 0.00 |
| Deinococcus | 0.00 | 0.00 | 0.00 | 0.00 | 0.00 | 0.00 | 0.00 | 0.00 | 0.00 |
| Deltaproteobacteria_unclassified | 0.00 | 0.00 | 0.00 | 0.00 | 0.00 | 0.00 | 0.00 | 0.00 | 0.00 |
| Denitromonas | 0.00 | 0.00 | 0.00 | 0.00 | 0.00 | 0.00 | 0.00 | 0.00 | 0.00 |
| Desulfarculaceae_uncultured | 0.00 | 0.00 | 0.00 | 0.00 | 0.00 | 0.00 | 0.00 | 0.00 | 0.00 |
| Desulfobacteraceae_unclassified | 0.00 | 0.00 | 0.00 | 0.00 | 0.00 | 0.00 | 0.00 | 0.00 | 0.00 |
| Desulfobacteraceae_uncultured | 0.00 | 0.00 | 0.00 | 0.00 | 0.00 | 0.00 | 0.00 | 0.00 | 0.00 |
| Desulfobulbaceae_unclassified | 0.00 | 0.00 | 0.00 | 0.00 | 0.00 | 0.00 | 0.00 | 0.00 | 0.00 |

| | | | | | | | | | |
|---|---|---|---|---|---|---|---|---|---|
| Desulfobulbaceae_uncultured | 0.03 | 0.07 | 0.04 | 0.01 | 0.00 | 0.01 | 0.00 | 0.00 | 0.00 |
| Desulfobulbus | 0.00 | 0.01 | 0.00 | 0.00 | 0.00 | 0.00 | 0.00 | 0.00 | 0.00 |
| Desulfococcus | 0.00 | 0.00 | 0.00 | 0.00 | 0.00 | 0.00 | 0.00 | 0.00 | 0.00 |
| Desulfomonile | 0.00 | 0.00 | 0.00 | 0.00 | 0.00 | 0.00 | 0.00 | 0.00 | 0.00 |
| Desulforhabdus | 0.00 | 0.00 | 0.00 | 0.00 | 0.00 | 0.00 | 0.00 | 0.00 | 0.00 |
| Desulforhopalus | 0.00 | 0.01 | 0.01 | 0.00 | 0.00 | 0.01 | 0.00 | 0.00 | 0.00 |
| Desulfosarcina | 0.00 | 0.00 | 0.00 | 0.00 | 0.00 | 0.00 | 0.00 | 0.00 | 0.00 |
| Dethiosulfatibacter | 0.00 | 0.00 | 0.00 | 0.00 | 0.00 | 0.00 | 0.00 | 0.00 | 0.00 |
| Devosia | 0.00 | 0.00 | 0.00 | 0.00 | 0.00 | 0.00 | 0.00 | 0.00 | 0.00 |
| Dietzia | 0.00 | 0.00 | 0.00 | 0.01 | 0.00 | 0.00 | 0.00 | 0.00 | 0.00 |
| Dokdonia | 0.01 | 0.01 | 0.01 | 0.02 | 0.01 | 0.01 | 0.00 | 0.00 | 0.01 |
| E01-9C-26_marine_group_norank | 0.01 | 0.01 | 0.00 | 0.00 | 0.00 | 0.00 | 0.01 | 0.00 | 0.00 |
| EC3_norank | 0.02 | 0.06 | 0.01 | 0.00 | 0.01 | 0.00 | 0.01 | 0.01 | 0.00 |
| EV818SWSAP88_norank | 0.00 | 0.01 | 0.00 | 0.00 | 0.00 | 0.00 | 0.00 | 0.00 | 0.00 |
| Ectothiorhodospiraceae_unclassified | 0.00 | 0.00 | 0.00 | 0.00 | 0.00 | 0.00 | 0.00 | 0.00 | 0.00 |
| Ekhidna | 0.01 | 0.01 | 0.00 | 0.00 | 0.00 | 0.00 | 0.00 | 0.01 | 0.00 |
| Elev-16S-1332_norank | 0.00 | 0.00 | 0.00 | 0.00 | 0.00 | 0.00 | 0.00 | 0.00 | 0.00 |
| Empedobacter | 0.00 | 0.00 | 0.01 | 0.01 | 0.01 | 0.00 | 0.00 | 0.00 | 0.01 |
| Endozoicomonas | 0.00 | 0.00 | 0.00 | 0.00 | 0.00 | 0.00 | 0.00 | 0.00 | 0.00 |
| Enhydrobacter | 0.04 | 0.05 | 0.11 | 0.03 | 0.06 | 0.05 | 0.05 | 0.03 | 0.05 |
| Enhygromyxa | 0.00 | 0.00 | 0.00 | 0.00 | 0.00 | 0.00 | 0.00 | 0.00 | 0.00 |

| | | | | | | | | | |
|---|---|---|---|---|---|---|---|---|---|
| Enterobacteriaceae_unclassified | 0.06 | 0.03 | 0.04 | 0.03 | 0.01 | 0.03 | 0.04 | 0.00 | 0.01 |
| Enterococcus | 0.04 | 0.01 | 0.06 | 0.01 | 0.03 | 0.03 | 0.02 | 0.04 | 0.04 |
| Epilithonimonas | 0.00 | 0.00 | 0.00 | 0.00 | 0.00 | 0.00 | 0.00 | 0.00 | 0.00 |
| Erysipelotrichaceae_uncultured | 0.00 | 0.05 | 0.00 | 0.00 | 0.00 | 0.00 | 0.01 | 0.01 | 0.00 |
| Erythrobacter | 0.35 | 0.37 | 0.26 | 0.23 | 0.23 | 0.19 | 0.11 | 0.03 | 0.05 |
| Erythrobacteraceae_unclassified | 0.18 | 0.13 | 0.09 | 0.02 | 0.01 | 0.03 | 0.02 | 0.02 | 0.01 |
| Eudoraea | 0.04 | 0.15 | 0.04 | 0.06 | 0.00 | 0.01 | 0.01 | 0.01 | 0.00 |
| Euzebyella | 0.00 | 0.00 | 0.00 | 0.00 | 0.01 | 0.00 | 0.00 | 0.00 | 0.00 |
| Exiguobacterium | 0.02 | 0.03 | 0.05 | 0.00 | 0.00 | 0.00 | 0.02 | 0.04 | 0.02 |
| FS117-23B-02_norank | 0.00 | 0.00 | 0.00 | 0.00 | 0.00 | 0.00 | 0.00 | 0.00 | 0.00 |
| Fabibacter | 0.00 | 0.00 | 0.00 | 0.00 | 0.00 | 0.00 | 0.00 | 0.00 | 0.00 |
| Facklamia | 0.00 | 0.00 | 0.00 | 0.00 | 0.00 | 0.00 | 0.00 | 0.00 | 0.00 |
| Family_XIII_uncultured | 0.04 | 0.01 | 0.00 | 0.00 | 0.00 | 0.00 | 0.00 | 0.00 | 0.00 |
| Family_XII_unclassified | 0.00 | 0.01 | 0.00 | 0.00 | 0.00 | 0.00 | 0.01 | 0.00 | 0.00 |
| Family_XI_uncultured | 0.00 | 0.05 | 0.00 | 0.00 | 0.00 | 0.00 | 0.00 | 0.01 | 0.01 |
| Ferrimonas | 0.06 | 0.02 | 0.01 | 0.02 | 0.01 | 0.06 | 0.01 | 0.04 | 0.03 |
| Fibrobacteraceae_uncultured | 0.00 | 0.00 | 0.01 | 0.00 | 0.00 | 0.00 | 0.00 | 0.00 | 0.00 |
| Filomicrobium | 0.06 | 0.10 | 0.02 | 0.01 | 0.01 | 0.02 | 0.01 | 0.00 | 0.01 |
| Flagellimonas | 0.11 | 0.20 | 0.19 | 0.31 | 0.22 | 0.34 | 0.03 | 0.01 | 0.03 |
| Flammeovirga | 0.00 | 0.00 | 0.00 | 0.00 | 0.00 | 0.00 | 0.00 | 0.00 | 0.00 |
| Flammeovirgaceae_unclassified | 0.01 | 0.00 | 0.00 | 0.00 | 0.00 | 0.00 | 0.00 | 0.00 | 0.00 |
| Flammeovirgaceae_uncultured | 0.02 | 0.13 | 0.06 | 0.01 | 0.00 | 0.02 | 0.00 | 0.01 | 0.02 |

| | | | | | | | | | |
|---|---|---|---|---|---|---|---|---|---|
| Flavobacteriaceae_unclassified | 2.46 | 5.23 | 2.36 | 6.34 | 4.21 | 5.68 | 0.86 | 0.75 | 2.24 |
| Flavobacteriaceae_uncultured | 0.41 | 0.91 | 0.86 | 2.47 | 3.07 | 1.22 | 0.51 | 0.63 | 1.28 |
| Flavobacteriales_unclassified | 0.00 | 0.00 | 0.00 | 0.08 | 0.05 | 0.00 | 0.00 | 0.00 | 0.01 |
| Flavobacterium | 0.45 | 0.04 | 0.26 | 1.91 | 1.94 | 1.16 | 0.09 | 0.04 | 0.23 |
| Fluviicola | 0.01 | 0.02 | 0.00 | 0.02 | 0.00 | 0.01 | 0.01 | 0.01 | 0.01 |
| Francisella | 0.00 | 0.00 | 0.00 | 0.00 | 0.00 | 0.00 | 0.00 | 0.00 | 0.00 |
| Fulvivirga | 0.02 | 0.05 | 0.05 | 0.01 | 0.01 | 0.01 | 0.01 | 0.01 | 0.01 |
| Fusibacter | 3.84 | 13.93 | 3.51 | 0.01 | 0.00 | 0.01 | 8.23 | 18.18 | 7.42 |
| Fusobacterium | 0.00 | 0.00 | 0.01 | 0.00 | 0.00 | 0.00 | 0.00 | 0.00 | 0.00 |
| GIF3_norank | 0.00 | 0.00 | 0.00 | 0.00 | 0.00 | 0.00 | 0.00 | 0.00 | 0.00 |
| GIF9_norank | 0.00 | 0.00 | 0.00 | 0.00 | 0.00 | 0.00 | 0.00 | 0.00 | 0.00 |
| GR-WP33-30_norank | 0.00 | 0.01 | 0.00 | 0.00 | 0.00 | 0.00 | 0.00 | 0.00 | 0.00 |
| GR-WP33-58_norank | 0.00 | 0.00 | 0.00 | 0.00 | 0.00 | 0.00 | 0.00 | 0.00 | 0.00 |
| Gaetbulibacter | 0.03 | 0.04 | 0.04 | 0.14 | 0.11 | 0.14 | 0.00 | 0.01 | 0.03 |
| Gaiella | 0.00 | 0.00 | 0.00 | 0.00 | 0.00 | 0.00 | 0.00 | 0.00 | 0.00 |
| Gaiellales_uncultured | 0.00 | 0.00 | 0.00 | 0.00 | 0.00 | 0.00 | 0.00 | 0.00 | 0.00 |
| Gammaproteobacteria_unclassified | 0.05 | 0.13 | 0.03 | 0.15 | 0.09 | 0.11 | 0.01 | 0.00 | 0.01 |
| Gangjinia | 1.71 | 0.06 | 2.83 | 0.66 | 0.82 | 0.89 | 0.77 | 0.06 | 6.08 |
| Gelidibacter | 0.00 | 0.00 | 0.00 | 0.00 | 0.00 | 0.00 | 0.00 | 0.00 | 0.00 |
| Gelria | 0.00 | 0.00 | 0.00 | 0.00 | 0.00 | 0.00 | 0.00 | 0.00 | 0.00 |
| Gemmatimonadaceae_uncultured | 0.00 | 0.00 | 0.00 | 0.00 | 0.00 | 0.00 | 0.00 | 0.00 | 0.00 |
| Geobacter | 0.00 | 0.00 | 0.00 | 0.00 | 0.00 | 0.00 | 0.00 | 0.00 | 0.00 |

| | | | | | | | | | |
|---|---|---|---|---|---|---|---|---|---|
| Gilvibacter | 0.00 | 0.01 | 0.00 | 0.01 | 0.00 | 0.00 | 0.00 | 0.00 | 0.01 |
| Gordonia | 0.05 | 0.01 | 0.01 | 0.01 | 0.02 | 0.02 | 0.00 | 0.00 | 0.00 |
| Gramella | 0.01 | 0.01 | 0.01 | 0.02 | 0.01 | 0.03 | 0.01 | 0.01 | 0.02 |
| Granulosicoccus | 0.10 | 0.08 | 0.07 | 0.01 | 0.01 | 0.02 | 0.01 | 0.01 | 0.01 |
| HOC36_norank | 0.00 | 0.00 | 0.00 | 0.00 | 0.00 | 0.00 | 0.00 | 0.00 | 0.00 |
| Haliangium | 0.00 | 0.00 | 0.00 | 0.00 | 0.00 | 0.00 | 0.00 | 0.00 | 0.00 |
| Haliea | 0.02 | 0.05 | 0.04 | 0.09 | 0.04 | 0.05 | 0.01 | 0.01 | 0.01 |
| Halioglobus | 0.00 | 0.00 | 0.00 | 0.02 | 0.01 | 0.02 | 0.01 | 0.00 | 0.00 |
| Haloferula | 0.00 | 0.00 | 0.00 | 0.00 | 0.00 | 0.00 | 0.00 | 0.00 | 0.00 |
| Halomonas | 0.39 | 0.24 | 0.11 | 0.04 | 0.04 | 0.03 | 0.02 | 0.00 | 0.00 |
| Haloplasma | 0.00 | 0.03 | 0.02 | 0.00 | 0.00 | 0.00 | 0.00 | 0.00 | 0.00 |
| Helcococcus | 0.00 | 0.00 | 0.00 | 0.00 | 0.00 | 0.00 | 0.00 | 0.00 | 0.00 |
| Hoeflea | 0.12 | 0.18 | 0.04 | 0.03 | 0.03 | 0.04 | 0.04 | 0.01 | 0.03 |
| Holosporaceae_uncultured | 0.00 | 0.00 | 0.00 | 0.00 | 0.00 | 0.00 | 0.00 | 0.00 | 0.00 |
| Hydrogenophaga | 0.00 | 0.00 | 0.00 | 0.00 | 0.00 | 0.00 | 0.00 | 0.00 | 0.00 |
| Hydrotalea | 0.00 | 0.00 | 0.00 | 0.00 | 0.00 | 0.00 | 0.00 | 0.00 | 0.00 |
| Hyphomicrobiaceae_unclassified | 0.01 | 0.01 | 0.01 | 0.00 | 0.00 | 0.00 | 0.00 | 0.00 | 0.00 |
| Hyphomicrobiaceae_uncultured | 0.00 | 0.02 | 0.00 | 0.00 | 0.00 | 0.00 | 0.00 | 0.00 | 0.00 |
| Hyphomonadaceae_unclassified | 0.00 | 0.00 | 0.00 | 0.00 | 0.00 | 0.00 | 0.00 | 0.00 | 0.00 |
| Hyphomonadaceae_uncultured | 0.03 | 0.03 | 0.00 | 0.05 | 0.02 | 0.00 | 0.00 | 0.00 | 0.00 |
| Hyphomonas | 0.00 | 0.00 | 0.00 | 0.00 | 0.00 | 0.01 | 0.00 | 0.00 | 0.00 |
| Idiomarina | 0.08 | 0.01 | 0.05 | 0.85 | 0.68 | 0.72 | 0.01 | 0.00 | 0.00 |

| | | | | | | | | | |
|---|---|---|---|---|---|---|---|---|---|
| Ignavibacterium | 0.00 | 0.00 | 0.00 | 0.00 | 0.00 | 0.00 | 0.00 | 0.00 | 0.00 |
| Ilumatobacter | 0.16 | 0.53 | 0.10 | 0.03 | 0.02 | 0.03 | 0.05 | 0.01 | 0.01 |
| JG30-KF-CM45_norank | 0.00 | 0.00 | 0.00 | 0.00 | 0.00 | 0.00 | 0.00 | 0.00 | 0.00 |
| JG30-KF-CM66_norank | 0.00 | 0.00 | 0.00 | 0.00 | 0.00 | 0.00 | 0.00 | 0.00 | 0.00 |
| JTB215_norank | 0.00 | 0.06 | 0.00 | 0.00 | 0.00 | 0.00 | 0.03 | 0.05 | 0.00 |
| JTB255_marine_benthic_group_norank | 0.23 | 0.30 | 0.14 | 0.05 | 0.06 | 0.08 | 0.03 | 0.06 | 0.02 |
| Janthinobacterium | 0.01 | 0.00 | 0.05 | 0.01 | 0.03 | 0.02 | 0.00 | 0.02 | 0.00 |
| KI89A_clade_norank | 0.01 | 0.02 | 0.00 | 0.01 | 0.00 | 0.00 | 0.00 | 0.00 | 0.00 |
| Kazan-2B-17_norank | 0.04 | 0.01 | 0.00 | 0.00 | 0.00 | 0.00 | 0.00 | 0.00 | 0.00 |
| Kordia | 0.00 | 0.00 | 0.00 | 0.01 | 0.02 | 0.02 | 0.00 | 0.00 | 0.00 |
| LD1-PA26_norank | 0.00 | 0.00 | 0.00 | 0.00 | 0.00 | 0.00 | 0.00 | 0.00 | 0.00 |
| LD1-PA34_norank | 0.00 | 0.01 | 0.00 | 0.00 | 0.00 | 0.00 | 0.00 | 0.00 | 0.00 |
| LD1-PB3_norank | 0.00 | 0.00 | 0.00 | 0.00 | 0.00 | 0.00 | 0.00 | 0.00 | 0.00 |
| LWSR-14_norank | 0.01 | 0.01 | 0.01 | 0.00 | 0.00 | 0.00 | 0.00 | 0.00 | 0.00 |
| Lachnospiraceae_unclassified | 0.00 | 0.04 | 0.00 | 0.00 | 0.00 | 0.00 | 0.00 | 0.01 | 0.00 |
| Lachnospiraceae_uncultured | 0.00 | 0.00 | 0.00 | 0.00 | 0.00 | 0.00 | 0.00 | 0.00 | 0.00 |
| Lactobacillus | 0.02 | 0.01 | 0.03 | 0.02 | 0.02 | 0.03 | 0.01 | 0.02 | 0.01 |
| Lactococcus | 3.16 | 1.12 | 6.61 | 3.50 | 3.75 | 3.68 | 3.04 | 2.81 | 2.46 |
| Leadbetterella | 0.00 | 0.00 | 0.00 | 0.00 | 0.00 | 0.00 | 0.00 | 0.00 | 0.00 |
| Legionella | 0.00 | 0.00 | 0.00 | 0.00 | 0.00 | 0.00 | 0.00 | 0.00 | 0.00 |
| Legionellaceae_unclassified | 0.01 | 0.02 | 0.02 | 0.00 | 0.00 | 0.01 | 0.00 | 0.00 | 0.00 |
| Legionellaceae_uncultured | 0.00 | 0.00 | 0.00 | 0.00 | 0.00 | 0.00 | 0.00 | 0.00 | 0.00 |

| | | | | | | | | | |
|---|---|---|---|---|---|---|---|---|---|
| Lentimonas | 0.01 | 0.01 | 0.00 | 0.00 | 0.00 | 0.00 | 0.00 | 0.00 | 0.00 |
| Lentisphaera | 0.00 | 0.00 | 0.00 | 0.00 | 0.00 | 0.00 | 0.00 | 0.00 | 0.00 |
| Lentisphaerae_unclassified | 0.00 | 0.00 | 0.00 | 0.00 | 0.00 | 0.00 | 0.00 | 0.00 | 0.00 |
| Leptobacterium | 0.02 | 0.02 | 0.07 | 0.00 | 0.00 | 0.00 | 0.01 | 0.00 | 0.01 |
| Leptolinea | 0.00 | 0.00 | 0.00 | 0.00 | 0.00 | 0.00 | 0.00 | 0.00 | 0.00 |
| Leptospiraceae_uncultured | 0.00 | 0.00 | 0.00 | 0.00 | 0.00 | 0.00 | 0.00 | 0.00 | 0.00 |
| Leptotrichiaceae_norank | 0.00 | 0.00 | 0.01 | 0.00 | 0.01 | 0.00 | 0.00 | 0.00 | 0.00 |
| Leptotrichiaceae_uncultured | 0.00 | 0.00 | 0.00 | 0.00 | 0.00 | 0.00 | 0.00 | 0.00 | 0.00 |
| Leucobacter | 0.00 | 0.00 | 0.00 | 0.00 | 0.00 | 0.00 | 0.00 | 0.00 | 0.00 |
| Leuconostoc | 0.11 | 0.03 | 0.22 | 0.04 | 0.07 | 0.10 | 0.06 | 0.12 | 0.06 |
| Leucothrix | 0.15 | 0.17 | 0.06 | 0.03 | 0.02 | 0.04 | 0.05 | 0.03 | 0.04 |
| Lewinella | 0.02 | 0.01 | 0.01 | 0.03 | 0.02 | 0.05 | 0.00 | 0.01 | 0.00 |
| Limibacter | 0.00 | 0.00 | 0.00 | 0.00 | 0.00 | 0.00 | 0.00 | 0.00 | 0.00 |
| Limnobacter | 0.00 | 0.00 | 0.00 | 0.00 | 0.00 | 0.00 | 0.00 | 0.00 | 0.00 |
| Lineage_I_(Endomicrobia)_norank | 0.00 | 0.00 | 0.00 | 0.00 | 0.00 | 0.00 | 0.00 | 0.00 | 0.00 |
| Loktanella | 0.01 | 0.06 | 0.01 | 0.01 | 0.00 | 0.00 | 0.00 | 0.01 | 0.01 |
| Longilinea | 0.00 | 0.00 | 0.00 | 0.00 | 0.00 | 0.00 | 0.00 | 0.00 | 0.00 |
| Luminiphilus | 0.00 | 0.00 | 0.00 | 0.00 | 0.00 | 0.00 | 0.00 | 0.00 | 0.00 |
| Luteivirga | 0.00 | 0.01 | 0.01 | 0.00 | 0.00 | 0.00 | 0.00 | 0.00 | 0.00 |
| Lutibacter | 0.26 | 6.47 | 0.45 | 1.41 | 0.79 | 1.90 | 0.86 | 0.82 | 0.64 |
| Lutispora | 0.00 | 0.00 | 0.00 | 0.00 | 0.00 | 0.00 | 0.00 | 0.00 | 0.00 |
| Lysobacter | 0.00 | 0.00 | 0.00 | 0.00 | 0.00 | 0.00 | 0.00 | 0.00 | 0.00 |

| | | | | | | | | | |
|---|---|---|---|---|---|---|---|---|---|
| M2PB4-65_termite_group_norank | 0.00 | 0.00 | 0.00 | 0.00 | 0.00 | 0.00 | 0.00 | 0.00 | 0.00 |
| MBAE14_norank | 0.00 | 0.00 | 0.00 | 0.03 | 0.00 | 0.00 | 0.00 | 0.00 | 0.00 |
| ML-AsS-8 | 0.00 | 0.01 | 0.00 | 0.00 | 0.00 | 0.00 | 0.00 | 0.00 | 0.00 |
| MSB-3A7_sediment_group_norank | 0.00 | 0.00 | 0.00 | 0.01 | 0.00 | 0.00 | 0.00 | 0.00 | 0.00 |
| Mangrovimonas | 0.11 | 0.00 | 0.19 | 0.04 | 0.08 | 0.07 | 0.00 | 0.00 | 0.01 |
| Maribacter | 0.23 | 0.31 | 0.13 | 0.23 | 0.10 | 0.29 | 0.15 | 0.05 | 0.13 |
| Marine_Methylotrophic_Group_3 | 0.00 | 0.00 | 0.00 | 0.00 | 0.00 | 0.00 | 0.00 | 0.00 | 0.00 |
| Marinicella | 0.21 | 0.60 | 0.19 | 0.24 | 0.09 | 0.10 | 0.02 | 0.04 | 0.03 |
| Marinifilum | 0.26 | 0.52 | 0.20 | 0.03 | 0.03 | 0.05 | 0.12 | 0.25 | 0.09 |
| Marinilabiaceae_uncultured | 0.01 | 0.54 | 0.12 | 0.01 | 0.01 | 0.02 | 0.11 | 0.48 | 0.09 |
| Marinobacter | 0.05 | 0.17 | 0.02 | 0.90 | 1.31 | 1.20 | 0.01 | 0.00 | 0.01 |
| Marinobacterium | 3.89 | 0.80 | 2.00 | 0.17 | 0.24 | 0.43 | 1.68 | 0.06 | 0.66 |
| Marinomonas | 0.38 | 0.31 | 0.13 | 0.26 | 0.29 | 0.39 | 0.92 | 0.22 | 1.19 |
| Marinosulfonomonas | 0.09 | 0.08 | 0.11 | 0.06 | 0.04 | 0.05 | 0.01 | 0.00 | 0.01 |
| Maritalea | 0.01 | 0.01 | 0.00 | 0.00 | 0.00 | 0.00 | 0.00 | 0.00 | 0.00 |
| Maritimimonas | 0.14 | 0.24 | 0.12 | 0.11 | 0.03 | 0.05 | 0.03 | 0.01 | 0.03 |
| Marivita | 0.14 | 0.18 | 0.08 | 0.04 | 0.02 | 0.04 | 0.05 | 0.02 | 0.02 |
| Marixanthomonas | 0.03 | 0.01 | 0.05 | 0.02 | 0.02 | 0.03 | 0.01 | 0.01 | 0.00 |
| Martelella | 0.00 | 0.00 | 0.00 | 0.00 | 0.00 | 0.00 | 0.00 | 0.00 | 0.00 |
| Mesoflavibacter | 2.98 | 0.46 | 1.88 | 2.41 | 1.86 | 2.42 | 0.67 | 0.12 | 8.00 |
| Mesonia | 0.00 | 0.01 | 0.03 | 0.05 | 0.07 | 0.08 | 0.00 | 0.00 | 0.00 |

| | | | | | | | | | |
|---|---|---|---|---|---|---|---|---|---|
| Methylobacterium | 0.00 | 0.00 | 0.00 | 0.00 | 0.00 | 0.00 | 0.00 | 0.00 | 0.00 |
| Methylocaldum | 0.00 | 0.00 | 0.00 | 0.00 | 0.00 | 0.00 | 0.00 | 0.00 | 0.00 |
| Methylophaga | 0.06 | 0.03 | 0.11 | 0.28 | 0.42 | 0.36 | 0.09 | 0.01 | 0.32 |
| Methylotenera | 0.26 | 0.21 | 0.16 | 0.09 | 0.13 | 0.09 | 0.08 | 0.04 | 0.06 |
| Microbacteriaceae_unclassified | 0.01 | 0.00 | 0.00 | 0.00 | 0.00 | 0.00 | 0.00 | 0.00 | 0.00 |
| Microbacterium | 0.00 | 0.00 | 0.00 | 0.00 | 0.00 | 0.00 | 0.00 | 0.00 | 0.00 |
| Microbulbifer | 0.00 | 0.00 | 0.01 | 0.00 | 0.00 | 0.01 | 0.00 | 0.00 | 0.00 |
| Milano-WF1B-44_norank | 0.00 | 0.00 | 0.00 | 0.00 | 0.00 | 0.00 | 0.00 | 0.00 | 0.00 |
| Mogibacterium | 0.00 | 0.00 | 0.00 | 0.00 | 0.00 | 0.00 | 0.00 | 0.00 | 0.00 |
| Mollicutes_unclassified | 0.00 | 0.01 | 0.00 | 0.00 | 0.00 | 0.00 | 0.00 | 0.01 | 0.01 |
| Moritella | 0.00 | 0.00 | 0.00 | 0.00 | 0.00 | 0.00 | 0.00 | 0.00 | 0.00 |
| Muricauda | 0.07 | 0.09 | 0.15 | 0.13 | 0.18 | 0.25 | 0.02 | 0.03 | 0.03 |
| Mycobacterium | 0.01 | 0.00 | 0.00 | 0.00 | 0.00 | 0.00 | 0.00 | 0.00 | 0.00 |
| Mycoplasma | 0.00 | 0.00 | 0.00 | 0.00 | 0.00 | 0.00 | 0.00 | 0.00 | 0.00 |
| Myroides | 0.07 | 0.01 | 0.18 | 0.06 | 0.04 | 0.08 | 0.09 | 0.11 | 0.05 |
| Myxococcales_unclassified | 0.00 | 0.00 | 0.00 | 0.00 | 0.00 | 0.00 | 0.00 | 0.00 | 0.00 |
| Myxococcales_uncultured | 0.00 | 0.00 | 0.00 | 0.00 | 0.00 | 0.00 | 0.00 | 0.00 | 0.00 |
| NB-1d_norank | 0.00 | 0.00 | 0.00 | 0.07 | 0.08 | 0.12 | 0.02 | 0.01 | 0.04 |
| NB1-n_norank | 0.00 | 0.03 | 0.00 | 0.00 | 0.00 | 0.00 | 0.03 | 0.10 | 0.05 |
| NKB5_norank | 0.00 | 0.00 | 0.00 | 0.00 | 0.00 | 0.00 | 0.01 | 0.00 | 0.00 |
| NS10_marine_group | 0.00 | 0.00 | 0.00 | 0.01 | 0.05 | 0.67 | 0.00 | 0.00 | 0.07 |

| | | | | | | | | | |
|---|---|---|---|---|---|---|---|---|---|
| NS11-12_marine_group_norank | 0.00 | 0.01 | 0.01 | 0.01 | 0.00 | 0.00 | 0.00 | 0.00 | 0.01 |
| NS3a_marine_group | 0.00 | 0.00 | 0.00 | 0.00 | 0.00 | 0.00 | 0.00 | 0.00 | 0.00 |
| NS4_marine_group | 0.00 | 0.00 | 0.00 | 0.00 | 0.00 | 0.00 | 0.00 | 0.00 | 0.00 |
| NS5_marine_group | 0.00 | 0.00 | 0.00 | 0.00 | 0.00 | 0.00 | 0.00 | 0.00 | 0.00 |
| NS72_norank | 0.00 | 0.00 | 0.00 | 0.00 | 0.00 | 0.00 | 0.00 | 0.00 | 0.00 |
| NS7_marine_group_norank | 0.01 | 0.00 | 0.00 | 0.00 | 0.00 | 0.00 | 0.00 | 0.00 | 0.00 |
| NS9_marine_group_norank | 0.00 | 0.02 | 0.01 | 0.02 | 0.00 | 0.01 | 0.00 | 0.00 | 0.00 |
| Nannocystaceae_unclassified | 0.00 | 0.00 | 0.00 | 0.00 | 0.00 | 0.00 | 0.00 | 0.00 | 0.00 |
| Nannocystaceae_uncultured | 0.00 | 0.00 | 0.00 | 0.00 | 0.00 | 0.00 | 0.00 | 0.00 | 0.00 |
| Nannocystis | 0.00 | 0.00 | 0.00 | 0.00 | 0.00 | 0.00 | 0.00 | 0.00 | 0.00 |
| Neptuniibacter | 0.00 | 0.00 | 0.00 | 0.00 | 0.00 | 0.00 | 0.01 | 0.00 | 0.22 |
| Neptunomonas | 1.31 | 0.33 | 0.01 | 0.08 | 0.09 | 0.05 | 5.95 | 1.39 | 3.48 |
| Nitratireductor | 0.00 | 0.00 | 0.00 | 0.01 | 0.01 | 0.00 | 0.00 | 0.00 | 0.00 |
| Nitrosomonadaceae_uncultured | 0.00 | 0.00 | 0.00 | 0.00 | 0.00 | 0.00 | 0.00 | 0.00 | 0.00 |
| Nitrosomonas | 0.00 | 0.00 | 0.00 | 0.00 | 0.00 | 0.00 | 0.00 | 0.00 | 0.00 |
| Nitrospinaceae_uncultured | 0.00 | 0.01 | 0.00 | 0.00 | 0.00 | 0.00 | 0.00 | 0.00 | 0.00 |
| Nitrospira | 0.00 | 0.00 | 0.00 | 0.00 | 0.00 | 0.00 | 0.00 | 0.00 | 0.00 |
| Nocardioides | 0.00 | 0.00 | 0.00 | 0.01 | 0.00 | 0.01 | 0.00 | 0.00 | 0.00 |
| Nonlabens | 0.07 | 0.01 | 0.03 | 0.09 | 0.08 | 0.08 | 0.00 | 0.00 | 0.03 |
| OCS116_clade_norank | 0.07 | 0.05 | 0.03 | 0.01 | 0.01 | 0.01 | 0.01 | 0.00 | 0.01 |
| OM190_norank | 0.04 | 0.16 | 0.04 | 0.01 | 0.02 | 0.01 | 0.01 | 0.01 | 0.01 |

| | | | | | | | | | |
|---|---|---|---|---|---|---|---|---|---|
| OM1_clade_norank | 0.05 | 0.09 | 0.02 | 0.00 | 0.00 | 0.00 | 0.01 | 0.01 | 0.00 |
| OM27_clade | 0.00 | 0.00 | 0.00 | 0.00 | 0.00 | 0.00 | 0.00 | 0.00 | 0.00 |
| OM60(NOR5)_clade | 0.03 | 0.10 | 0.04 | 0.03 | 0.02 | 0.02 | 0.01 | 0.01 | 0.03 |
| OPB56_norank | 0.03 | 0.05 | 0.02 | 0.03 | 0.02 | 0.01 | 0.01 | 0.01 | 0.02 |
| Oceanicella | 0.00 | 0.00 | 0.00 | 0.00 | 0.00 | 0.00 | 0.00 | 0.00 | 0.00 |
| Oceanimonas | 0.00 | 0.21 | 0.01 | 0.00 | 0.00 | 0.00 | 0.07 | 0.05 | 0.15 |
| Oceaniovalibus | 0.01 | 0.01 | 0.01 | 0.00 | 0.00 | 0.01 | 0.00 | 0.00 | 0.00 |
| Oceanisphaera | 0.00 | 0.14 | 0.00 | 0.00 | 0.00 | 0.00 | 0.00 | 0.00 | 0.00 |
| Oceanospirillaceae_unclassified | 0.05 | 0.00 | 0.09 | 0.59 | 1.17 | 0.70 | 0.00 | 0.00 | 0.01 |
| Ochrobactrum | 0.00 | 0.00 | 0.00 | 0.00 | 0.00 | 0.00 | 0.00 | 0.00 | 0.00 |
| Oleispira | 0.01 | 0.00 | 0.07 | 1.06 | 0.76 | 0.95 | 0.04 | 0.00 | 0.00 |
| Oligosphaerales_norank | 0.00 | 0.00 | 0.00 | 0.00 | 0.00 | 0.00 | 0.00 | 0.00 | 0.00 |
| Opitutae_unclassified | 0.00 | 0.00 | 0.00 | 0.00 | 0.00 | 0.00 | 0.00 | 0.00 | 0.00 |
| Ornatilinea | 0.00 | 0.00 | 0.00 | 0.00 | 0.00 | 0.00 | 0.00 | 0.00 | 0.00 |
| Ornithinimicrobium | 0.00 | 0.00 | 0.00 | 0.00 | 0.00 | 0.00 | 0.00 | 0.00 | 0.00 |
| Ornithobacterium | 0.00 | 0.00 | 0.00 | 0.00 | 0.00 | 0.00 | 0.00 | 0.00 | 0.00 |
| Owenweeksia | 0.01 | 0.02 | 0.07 | 0.50 | 0.46 | 0.81 | 0.03 | 0.00 | 0.22 |
| PAUC26f_norank | 0.00 | 0.00 | 0.00 | 0.00 | 0.00 | 0.00 | 0.00 | 0.00 | 0.00 |
| PAUC43f_marine_benthic_group_norank | 0.00 | 0.02 | 0.00 | 0.00 | 0.00 | 0.00 | 0.00 | 0.00 | 0.00 |
| PHOS-HE36_norank | 0.00 | 0.00 | 0.00 | 0.00 | 0.00 | 0.00 | 0.00 | 0.00 | 0.00 |
| PHOS-HE51_norank | 0.00 | 0.00 | 0.00 | 0.00 | 0.00 | 0.00 | 0.00 | 0.00 | 0.00 |

| | | | | | | | | | |
|---|---|---|---|---|---|---|---|---|---|
| Paenibacillaceae_unclassified | 0.00 | 0.00 | 0.00 | 0.00 | 0.00 | 0.00 | 0.00 | 0.00 | 0.00 |
| Paenibacillus | 0.00 | 0.00 | 0.00 | 0.00 | 0.00 | 0.00 | 0.00 | 0.00 | 0.00 |
| Paracoccus | 0.06 | 0.04 | 0.05 | 0.05 | 0.12 | 0.06 | 0.01 | 0.00 | 0.02 |
| Paramoritella | 0.00 | 0.00 | 0.00 | 0.00 | 0.00 | 0.00 | 0.00 | 0.00 | 0.00 |
| Parasphingopyxis | 0.01 | 0.01 | 0.01 | 0.00 | 0.00 | 0.00 | 0.00 | 0.00 | 0.00 |
| Parvularcula | 0.02 | 0.01 | 0.01 | 0.01 | 0.00 | 0.00 | 0.00 | 0.00 | 0.01 |
| PeM15_norank | 0.01 | 0.02 | 0.02 | 0.00 | 0.00 | 0.01 | 0.00 | 0.00 | 0.00 |
| Pedobacter | 0.00 | 0.00 | 0.00 | 0.00 | 0.01 | 0.00 | 0.00 | 0.00 | 0.00 |
| Pelagibacterium | 0.00 | 0.03 | 0.00 | 0.01 | 0.00 | 0.00 | 0.00 | 0.01 | 0.01 |
| Peptostreptococcaceae_incertae_sedis | 0.04 | 0.21 | 0.07 | 0.00 | 0.00 | 0.01 | 0.03 | 0.37 | 0.00 |
| Peredibacter | 0.00 | 0.01 | 0.00 | 0.19 | 0.14 | 0.00 | 0.00 | 0.00 | 0.00 |
| Perlucidibaca | 0.00 | 0.00 | 0.01 | 0.00 | 0.00 | 0.00 | 0.00 | 0.00 | 0.00 |
| Persicirhabdus | 0.02 | 0.01 | 0.01 | 0.00 | 0.00 | 0.00 | 0.00 | 0.01 | 0.00 |
| Persicobacter | 0.04 | 0.06 | 0.11 | 0.00 | 0.01 | 0.00 | 0.00 | 0.02 | 0.04 |
| Phaeobacter | 0.38 | 0.10 | 0.09 | 0.11 | 0.10 | 0.31 | 0.02 | 0.01 | 0.03 |
| Phascolarctobacterium | 0.00 | 0.00 | 0.00 | 0.00 | 0.00 | 0.00 | 0.00 | 0.00 | 0.00 |
| Phormidium | 0.00 | 0.00 | 0.00 | 0.00 | 0.00 | 0.00 | 0.00 | 0.00 | 0.00 |
| Photobacterium | 0.02 | 0.11 | 0.01 | 0.02 | 0.01 | 0.01 | 0.01 | 0.01 | 0.16 |
| Phycisphaera | 0.00 | 0.01 | 0.01 | 0.00 | 0.01 | 0.00 | 0.00 | 0.00 | 0.00 |
| Phyllobacteriaceae_unclassified | 0.02 | 0.03 | 0.02 | 0.01 | 0.01 | 0.01 | 0.01 | 0.01 | 0.00 |
| Phyllobacteriaceae_uncultured | 0.07 | 0.06 | 0.04 | 0.00 | 0.00 | 0.00 | 0.08 | 0.02 | 0.03 |
| Pir4_lineage | 0.06 | 0.14 | 0.04 | 0.01 | 0.00 | 0.01 | 0.02 | 0.01 | 0.01 |

| | | | | | | | | | |
|---|---|---|---|---|---|---|---|---|---|
| Pirellula | 0.07 | 0.18 | 0.05 | 0.03 | 0.02 | 0.02 | 0.03 | 0.01 | 0.01 |
| Piscirickettsiaceae_unclassified | 0.01 | 0.01 | 0.02 | 0.05 | 0.05 | 0.04 | 0.01 | 0.00 | 0.04 |
| Piscirickettsiaceae_uncultured | 0.00 | 0.00 | 0.00 | 0.01 | 0.02 | 0.00 | 0.00 | 0.00 | 0.00 |
| Planctomyces | 0.36 | 0.78 | 0.31 | 0.09 | 0.06 | 0.09 | 0.18 | 0.17 | 0.10 |
| Planctomycetaceae_unclassified | 0.03 | 0.05 | 0.02 | 0.00 | 0.00 | 0.00 | 0.01 | 0.01 | 0.00 |
| Planctomycetaceae_uncultured | 0.00 | 0.01 | 0.02 | 0.00 | 0.00 | 0.00 | 0.00 | 0.00 | 0.00 |
| Planctomycetes_unclassified | 0.00 | 0.00 | 0.00 | 0.00 | 0.00 | 0.00 | 0.00 | 0.00 | 0.00 |
| Planktotalea | 1.35 | 2.36 | 1.84 | 0.87 | 0.87 | 1.00 | 0.35 | 0.23 | 0.39 |
| Planococcaceae_unclassified | 0.02 | 0.05 | 0.05 | 0.03 | 0.04 | 0.04 | 0.05 | 0.03 | 0.02 |
| Pleurocapsa | 0.02 | 0.02 | 0.02 | 0.00 | 0.00 | 0.00 | 0.01 | 0.00 | 0.00 |
| Polaribacter | 0.04 | 0.06 | 0.02 | 0.09 | 0.13 | 0.16 | 0.02 | 0.03 | 0.03 |
| Polaromonas | 0.00 | 0.00 | 0.00 | 0.00 | 0.00 | 0.00 | 0.00 | 0.00 | 0.00 |
| Porphyromonadaceae_uncultured | 0.00 | 0.01 | 0.00 | 0.00 | 0.00 | 0.00 | 0.00 | 0.00 | 0.00 |
| Porphyromonas | 0.00 | 0.00 | 0.00 | 0.00 | 0.00 | 0.00 | 0.01 | 0.00 | 0.00 |
| Portibacter | 0.01 | 0.04 | 0.01 | 0.00 | 0.00 | 0.00 | 0.00 | 0.02 | 0.01 |
| Porticoccus | 0.00 | 0.00 | 0.00 | 0.00 | 0.00 | 0.00 | 0.00 | 0.00 | 0.00 |
| Prevotella | 0.00 | 0.00 | 0.00 | 0.00 | 0.00 | 0.00 | 0.00 | 0.00 | 0.00 |
| Prevotellaceae_uncultured | 0.00 | 0.00 | 0.00 | 0.00 | 0.00 | 0.00 | 0.00 | 0.00 | 0.00 |
| Profundibacterium | 0.09 | 0.28 | 0.07 | 0.02 | 0.01 | 0.01 | 0.02 | 0.01 | 0.01 |
| Prolixibacter | 0.00 | 0.01 | 0.00 | 0.00 | 0.00 | 0.00 | 0.00 | 0.47 | 0.00 |
| Propionibacteriaceae_uncultured | 0.00 | 0.00 | 0.00 | 0.00 | 0.00 | 0.00 | 0.00 | 0.00 | 0.00 |
| Propionibacterium | 0.00 | 0.00 | 0.00 | 0.00 | 0.00 | 0.00 | 0.00 | 0.00 | 0.00 |

| | | | | | | | | | |
|---|---|---|---|---|---|---|---|---|---|
| Propioniciclava | 0.00 | 0.00 | 0.00 | 0.00 | 0.00 | 0.00 | 0.00 | 0.00 | 0.00 |
| Propionigenium | 0.19 | 0.37 | 0.01 | 0.01 | 0.00 | 0.00 | 0.02 | 0.01 | 0.01 |
| Proteiniclasticum | 0.78 | 2.80 | 0.54 | 0.01 | 0.00 | 0.01 | 1.22 | 0.65 | 0.24 |
| Proteobacteria_unclassified | 0.00 | 0.00 | 0.00 | 0.00 | 0.00 | 0.00 | 0.00 | 0.00 | 0.00 |
| Pseudahrensia | 0.08 | 0.13 | 0.05 | 0.01 | 0.02 | 0.01 | 0.08 | 0.06 | 0.02 |
| Pseudoalteromonadaceae_uncultured | 0.00 | 0.00 | 0.00 | 0.02 | 0.02 | 0.01 | 0.00 | 0.00 | 0.00 |
| Pseudoalteromonas | 21.83 | 8.40 | 22.92 | 20.13 | 24.97 | 15.92 | 9.91 | 6.95 | 13.28 |
| Pseudolabrys | 0.00 | 0.00 | 0.00 | 0.00 | 0.00 | 0.00 | 0.00 | 0.00 | 0.00 |
| Pseudomaricurvus | 0.00 | 0.00 | 0.00 | 0.01 | 0.01 | 0.00 | 0.00 | 0.00 | 0.00 |
| Pseudomonas | 0.60 | 0.23 | 1.03 | 0.64 | 0.67 | 0.53 | 0.72 | 0.40 | 0.45 |
| Pseudonocardia | 0.01 | 0.00 | 0.00 | 0.00 | 0.01 | 0.00 | 0.00 | 0.00 | 0.00 |
| Pseudorhodobacter | 0.00 | 0.00 | 0.00 | 0.00 | 0.00 | 0.00 | 0.00 | 0.00 | 0.00 |
| Pseudovibrio | 0.23 | 0.01 | 0.11 | 0.00 | 0.00 | 0.00 | 0.00 | 0.00 | 0.00 |
| Pseudoxanthomonas | 0.00 | 0.00 | 0.00 | 0.00 | 0.00 | 0.00 | 0.00 | 0.00 | 0.00 |
| Psychrilyobacter | 0.00 | 0.01 | 0.00 | 0.00 | 0.00 | 0.00 | 0.00 | 0.00 | 0.00 |
| Psychrobacter | 0.20 | 1.60 | 0.38 | 0.40 | 0.38 | 0.32 | 0.23 | 0.07 | 0.23 |
| Psychroflexus | 0.01 | 0.00 | 0.02 | 0.01 | 0.01 | 0.02 | 0.04 | 0.00 | 0.26 |
| Psychromonas | 0.00 | 0.02 | 0.03 | 0.01 | 0.00 | 0.04 | 0.01 | 0.01 | 0.00 |
| Psychroserpens | 0.56 | 0.46 | 0.54 | 1.08 | 0.77 | 1.08 | 0.26 | 0.14 | 0.61 |
| R76-B128_norank | 0.00 | 0.00 | 0.00 | 0.00 | 0.00 | 0.00 | 0.00 | 0.00 | 0.00 |
| RB41_norank | 0.00 | 0.00 | 0.00 | 0.00 | 0.00 | 0.00 | 0.00 | 0.00 | 0.00 |
| RF9_norank | 0.00 | 0.00 | 0.00 | 0.00 | 0.00 | 0.00 | 0.00 | 0.03 | 0.00 |

| | | | | | | | | | |
|---|---|---|---|---|---|---|---|---|---|
| Rahnella | 0.02 | 0.02 | 0.04 | 0.04 | 0.03 | 0.02 | 0.04 | 0.02 | 0.01 |
| Ralstonia | 0.00 | 0.00 | 0.00 | 0.00 | 0.00 | 0.00 | 0.00 | 0.00 | 0.00 |
| Reichenbachiella | 0.00 | 0.01 | 0.01 | 0.01 | 0.00 | 0.00 | 0.00 | 0.00 | 0.00 |
| Reinekea | 0.00 | 0.00 | 0.00 | 0.01 | 0.00 | 0.00 | 0.00 | 0.00 | 0.00 |
| Rhizobiaceae_unclassified | 0.00 | 0.00 | 0.00 | 0.00 | 0.00 | 0.00 | 0.00 | 0.00 | 0.00 |
| Rhizobiales_unclassified | 0.00 | 0.01 | 0.00 | 0.00 | 0.00 | 0.00 | 0.00 | 0.00 | 0.00 |
| Rhizobiales_uncultured | 0.00 | 0.00 | 0.00 | 0.00 | 0.00 | 0.00 | 0.00 | 0.00 | 0.00 |
| Rhodobacteraceae_unclassified | 1.62 | 3.75 | 1.49 | 1.00 | 0.73 | 0.78 | 0.28 | 0.24 | 0.25 |
| Rhodobacteraceae_uncultured | 0.03 | 0.07 | 0.03 | 0.01 | 0.01 | 0.00 | 0.01 | 0.00 | 0.00 |
| Rhodobium | 0.02 | 0.05 | 0.00 | 0.02 | 0.00 | 0.02 | 0.00 | 0.00 | 0.01 |
| Rhodococcus | 0.00 | 0.00 | 0.00 | 0.00 | 0.00 | 0.00 | 0.00 | 0.00 | 0.00 |
| Rhodopirellula | 0.31 | 0.92 | 0.32 | 0.10 | 0.07 | 0.05 | 0.10 | 0.14 | 0.07 |
| Rhodospirillaceae_uncultured | 0.01 | 0.01 | 0.01 | 0.00 | 0.00 | 0.00 | 0.00 | 0.00 | 0.00 |
| Rhodospirillales_unclassified | 0.00 | 0.00 | 0.00 | 0.00 | 0.00 | 0.00 | 0.00 | 0.00 | 0.00 |
| Rhodothermaceae_uncultured | 0.00 | 0.01 | 0.00 | 0.00 | 0.00 | 0.00 | 0.00 | 0.00 | 0.00 |
| Rhodovulum | 0.00 | 0.00 | 0.00 | 0.00 | 0.00 | 0.00 | 0.00 | 0.00 | 0.00 |
| Rickettsia | 0.01 | 0.01 | 0.00 | 0.00 | 0.00 | 0.00 | 0.00 | 0.00 | 0.00 |
| Rickettsiaceae_uncultured | 0.00 | 0.00 | 0.00 | 0.00 | 0.00 | 0.00 | 0.00 | 0.00 | 0.00 |
| Rickettsiales_unclassified | 0.00 | 0.00 | 0.00 | 0.00 | 0.00 | 0.00 | 0.00 | 0.00 | 0.00 |
| Rivularia | 0.00 | 0.00 | 0.00 | 0.00 | 0.00 | 0.00 | 0.00 | 0.00 | 0.00 |
| Robiginitalea | 0.00 | 0.02 | 0.01 | 0.01 | 0.00 | 0.01 | 0.00 | 0.01 | 0.00 |
| Robiginitomaculum | 0.00 | 0.00 | 0.00 | 0.00 | 0.00 | 0.00 | 0.00 | 0.00 | 0.00 |

| | | | | | | | | | |
|---|---|---|---|---|---|---|---|---|---|
| Roseburia | 0.00 | 0.00 | 0.00 | 0.00 | 0.00 | 0.00 | 0.00 | 0.00 | 0.00 |
| Roseibacillus | 0.00 | 0.00 | 0.00 | 0.00 | 0.00 | 0.00 | 0.00 | 0.00 | 0.00 |
| Roseobacter_clade_NAC11-7_lineage | 0.10 | 0.14 | 0.07 | 0.05 | 0.01 | 0.03 | 0.02 | 0.01 | 0.01 |
| Roseomonas | 0.00 | 0.00 | 0.00 | 0.00 | 0.00 | 0.00 | 0.00 | 0.00 | 0.00 |
| Roseovarius | 0.01 | 0.00 | 0.00 | 0.00 | 0.00 | 0.00 | 0.00 | 0.00 | 0.00 |
| Rubidimonas | 0.00 | 0.00 | 0.00 | 0.00 | 0.00 | 0.00 | 0.00 | 0.00 | 0.00 |
| Rubricoccus | 0.01 | 0.01 | 0.00 | 0.00 | 0.01 | 0.00 | 0.00 | 0.00 | 0.00 |
| Rubritalea | 0.00 | 0.00 | 0.00 | 0.00 | 0.00 | 0.00 | 0.00 | 0.00 | 0.00 |
| Rubrivirga | 0.02 | 0.02 | 0.02 | 0.07 | 0.03 | 0.04 | 0.00 | 0.01 | 0.00 |
| Ruegeria | 0.92 | 2.80 | 1.51 | 0.93 | 0.61 | 0.65 | 0.18 | 0.15 | 0.13 |
| Ruminococcaceae_unclassified | 0.00 | 0.00 | 0.00 | 0.00 | 0.00 | 0.00 | 0.00 | 0.00 | 0.00 |
| Ruminococcaceae_uncultured | 0.00 | 0.00 | 0.01 | 0.00 | 0.00 | 0.00 | 0.00 | 0.00 | 0.00 |
| Run-SP154_norank | 0.00 | 0.00 | 0.00 | 0.00 | 0.00 | 0.00 | 0.00 | 0.00 | 0.00 |
| SAR324_clade(Marine_group_B)_norank | 0.01 | 0.00 | 0.00 | 0.00 | 0.00 | 0.00 | 0.00 | 0.00 | 0.00 |
| SB-1_norank | 0.02 | 0.42 | 0.06 | 0.02 | 0.00 | 0.01 | 0.00 | 0.10 | 0.00 |
| SB-5_norank | 0.00 | 0.00 | 0.00 | 0.00 | 0.00 | 0.00 | 0.00 | 0.00 | 0.00 |
| SB1-18_norank | 0.00 | 0.00 | 0.00 | 0.01 | 0.00 | 0.00 | 0.00 | 0.00 | 0.00 |
| SC3-20_norank | 0.01 | 0.01 | 0.00 | 0.01 | 0.00 | 0.00 | 0.00 | 0.00 | 0.01 |
| SEEP-SRB1 | 0.00 | 0.00 | 0.00 | 0.00 | 0.00 | 0.00 | 0.00 | 0.00 | 0.00 |
| SHA-109_norank | 0.02 | 0.01 | 0.01 | 0.00 | 0.00 | 0.00 | 0.02 | 0.00 | 0.01 |
| SJA-28_norank | 0.00 | 0.00 | 0.00 | 0.00 | 0.00 | 0.00 | 0.00 | 0.00 | 0.00 |

| | | | | | | | | | |
|---|---|---|---|---|---|---|---|---|---|
| SM1A02 | 0.00 | 0.01 | 0.00 | 0.01 | 0.00 | 0.01 | 0.01 | 0.00 | 0.00 |
| SM2D12_norank | 0.00 | 0.00 | 0.00 | 0.00 | 0.00 | 0.00 | 0.00 | 0.00 | 0.00 |
| SPOTSOCT00m83_norank | 0.00 | 0.00 | 0.00 | 0.00 | 0.00 | 0.00 | 0.00 | 0.00 | 0.00 |
| SS1-B-03-39_norank | 0.00 | 0.01 | 0.00 | 0.00 | 0.00 | 0.00 | 0.00 | 0.00 | 0.00 |
| SS1-B-06-26_norank | 0.01 | 0.03 | 0.01 | 0.01 | 0.00 | 0.01 | 0.01 | 0.00 | 0.00 |
| SWB04_norank | 0.01 | 0.02 | 0.01 | 0.01 | 0.00 | 0.01 | 0.00 | 0.00 | 0.00 |
| SZB30_norank | 0.00 | 0.00 | 0.00 | 0.00 | 0.00 | 0.00 | 0.00 | 0.00 | 0.00 |
| Salegentibacter | 0.40 | 0.23 | 0.38 | 0.48 | 0.37 | 0.62 | 0.25 | 0.21 | 0.66 |
| Salinicoccus | 0.00 | 0.00 | 0.01 | 0.00 | 0.00 | 0.00 | 0.01 | 0.00 | 0.01 |
| Salinimicrobium | 0.16 | 0.03 | 0.11 | 0.00 | 0.02 | 0.03 | 0.06 | 0.01 | 0.03 |
| Salinirepens | 0.01 | 0.06 | 0.00 | 0.29 | 0.12 | 0.03 | 0.00 | 0.01 | 0.03 |
| Sandaracinaceae_norank | 0.02 | 0.04 | 0.00 | 0.01 | 0.01 | 0.01 | 0.00 | 0.00 | 0.00 |
| Sandaracinaceae_unclassified | 0.00 | 0.00 | 0.00 | 0.00 | 0.00 | 0.00 | 0.00 | 0.00 | 0.00 |
| Sandaracinus | 0.00 | 0.00 | 0.00 | 0.00 | 0.00 | 0.00 | 0.00 | 0.00 | 0.00 |
| Saprospira | 0.00 | 0.00 | 0.00 | 0.00 | 0.00 | 0.00 | 0.00 | 0.00 | 0.00 |
| Saprospiraceae_unclassified | 0.00 | 0.01 | 0.01 | 0.00 | 0.00 | 0.00 | 0.00 | 0.01 | 0.00 |
| Saprospiraceae_uncultured | 0.14 | 0.23 | 0.10 | 0.07 | 0.03 | 0.07 | 0.02 | 0.03 | 0.00 |
| Schleiferia | 0.00 | 0.00 | 0.00 | 0.00 | 0.00 | 0.00 | 0.00 | 0.00 | 0.00 |
| Sedimentibacter | 0.00 | 0.01 | 0.00 | 0.00 | 0.00 | 0.00 | 0.00 | 0.00 | 0.00 |
| Sediminibacter | 0.00 | 0.00 | 0.00 | 0.00 | 0.00 | 0.00 | 0.00 | 0.00 | 0.00 |
| Sh765B-TzT-29_norank | 0.02 | 0.03 | 0.02 | 0.01 | 0.00 | 0.01 | 0.00 | 0.01 | 0.00 |
| Shewanella | 0.31 | 0.29 | 0.10 | 0.68 | 0.91 | 1.59 | 0.07 | 0.03 | 0.04 |

| | | | | | | | | | |
|---|---|---|---|---|---|---|---|---|---|
| Simiduia | 0.00 | 0.00 | 0.00 | 0.00 | 0.00 | 0.00 | 0.00 | 0.00 | 0.00 |
| Skagenf62_norank | 0.00 | 0.00 | 0.00 | 0.00 | 0.00 | 0.00 | 0.01 | 0.00 | 0.01 |
| Smithella | 0.00 | 0.00 | 0.00 | 0.00 | 0.00 | 0.00 | 0.00 | 0.00 | 0.00 |
| Solibacillus | 0.05 | 0.00 | 0.06 | 0.01 | 0.05 | 0.06 | 0.04 | 0.03 | 0.03 |
| Solirubrobacter | 0.00 | 0.00 | 0.00 | 0.00 | 0.00 | 0.00 | 0.00 | 0.00 | 0.00 |
| Sphingobacteriales_unclassified | 0.00 | 0.00 | 0.00 | 0.00 | 0.00 | 0.00 | 0.00 | 0.00 | 0.00 |
| Sphingobacterium | 0.00 | 0.01 | 0.01 | 0.01 | 0.00 | 0.00 | 0.00 | 0.00 | 0.01 |
| Sphingobium | 0.00 | 0.00 | 0.00 | 0.00 | 0.00 | 0.00 | 0.00 | 0.00 | 0.00 |
| Sphingomicrobium | 0.00 | 0.01 | 0.00 | 0.00 | 0.00 | 0.00 | 0.00 | 0.00 | 0.00 |
| Sphingomonadaceae_unclassified | 0.00 | 0.00 | 0.00 | 0.00 | 0.00 | 0.00 | 0.00 | 0.00 | 0.00 |
| Sphingomonadaceae_uncultured | 0.00 | 0.00 | 0.00 | 0.00 | 0.00 | 0.00 | 0.00 | 0.00 | 0.00 |
| Sphingomonadales_unclassified | 0.01 | 0.01 | 0.01 | 0.01 | 0.01 | 0.00 | 0.02 | 0.00 | 0.00 |
| Sphingomonas | 0.00 | 0.00 | 0.00 | 0.00 | 0.00 | 0.00 | 0.00 | 0.00 | 0.00 |
| Sphingorhabdus | 0.00 | 0.02 | 0.02 | 0.02 | 0.03 | 0.01 | 0.01 | 0.01 | 0.01 |
| Spirochaeta | 0.00 | 0.00 | 0.00 | 0.00 | 0.00 | 0.00 | 0.00 | 0.00 | 0.00 |
| Spirochaetaceae_uncultured | 0.00 | 0.00 | 0.00 | 0.00 | 0.00 | 0.00 | 0.00 | 0.00 | 0.00 |
| Spongiibacter | 0.00 | 0.00 | 0.00 | 0.00 | 0.00 | 0.00 | 0.00 | 0.00 | 0.00 |
| Staphylococcus | 0.00 | 0.00 | 0.01 | 0.00 | 0.00 | 0.00 | 0.00 | 0.00 | 0.00 |
| Stappia | 0.00 | 0.00 | 0.00 | 0.00 | 0.00 | 0.01 | 0.00 | 0.00 | 0.00 |
| Stenotrophomonas | 0.00 | 0.00 | 0.01 | 0.00 | 0.01 | 0.00 | 0.01 | 0.00 | 0.01 |
| Streptococcus | 0.12 | 0.12 | 0.22 | 0.11 | 0.09 | 0.09 | 0.10 | 0.10 | 0.08 |
| Streptomyces | 0.01 | 0.00 | 0.00 | 0.00 | 0.00 | 0.00 | 0.00 | 0.00 | 0.00 |

| | | | | | | | | | |
|---|---|---|---|---|---|---|---|---|---|
| Subgroup_17_norank | 0.00 | 0.01 | 0.00 | 0.00 | 0.00 | 0.00 | 0.00 | 0.00 | 0.00 |
| Subgroup_21_norank | 0.00 | 0.00 | 0.00 | 0.00 | 0.00 | 0.00 | 0.00 | 0.00 | 0.00 |
| Subgroup_23_norank | 0.01 | 0.03 | 0.00 | 0.00 | 0.00 | 0.01 | 0.00 | 0.00 | 0.00 |
| Subgroup_6_norank | 0.00 | 0.00 | 0.00 | 0.00 | 0.00 | 0.00 | 0.00 | 0.00 | 0.00 |
| Subgroup_9_norank | 0.00 | 0.01 | 0.01 | 0.00 | 0.00 | 0.00 | 0.00 | 0.00 | 0.00 |
| SubsectionI_FamilyI_norank | 0.00 | 0.00 | 0.00 | 0.00 | 0.00 | 0.00 | 0.00 | 0.00 | 0.00 |
| SubsectionI_FamilyI_unclassified | 0.00 | 0.00 | 0.00 | 0.00 | 0.00 | 0.00 | 0.00 | 0.00 | 0.00 |
| SubsectionI_FamilyI_uncultured | 0.02 | 0.01 | 0.01 | 0.00 | 0.00 | 0.00 | 0.00 | 0.01 | 0.00 |
| Sulfitobacter | 0.31 | 0.44 | 0.18 | 0.23 | 0.21 | 0.22 | 0.07 | 0.04 | 0.04 |
| Sulfurovum | 0.04 | 0.11 | 0.02 | 0.01 | 0.00 | 0.01 | 0.00 | 0.01 | 0.01 |
| Sunxiuqinia | 0.52 | 0.62 | 0.60 | 0.00 | 0.02 | 0.01 | 0.16 | 0.50 | 0.06 |
| Surface_1_norank | 0.00 | 0.00 | 0.00 | 0.00 | 0.00 | 0.00 | 0.00 | 0.00 | 0.00 |
| Sva0071_norank | 0.03 | 0.05 | 0.01 | 0.01 | 0.00 | 0.00 | 0.01 | 0.00 | 0.00 |
| Sva0081_sediment_group | 0.00 | 0.01 | 0.00 | 0.01 | 0.00 | 0.00 | 0.00 | 0.00 | 0.00 |
| Sva0485_norank | 0.00 | 0.00 | 0.00 | 0.00 | 0.00 | 0.00 | 0.00 | 0.00 | 0.00 |
| Sva0725_norank | 0.02 | 0.06 | 0.01 | 0.00 | 0.00 | 0.00 | 0.00 | 0.01 | 0.01 |
| Sva0996_marine_group_norank | 0.17 | 0.25 | 0.05 | 0.04 | 0.01 | 0.02 | 0.01 | 0.01 | 0.01 |
| Sva1033_norank | 0.00 | 0.01 | 0.00 | 0.00 | 0.00 | 0.00 | 0.00 | 0.00 | 0.00 |
| Synechococcus | 0.02 | 0.06 | 0.02 | 0.01 | 0.00 | 0.00 | 0.01 | 0.00 | 0.00 |
| Synergistaceae_uncultured | 0.00 | 0.00 | 0.00 | 0.00 | 0.00 | 0.00 | 0.00 | 0.00 | 0.00 |
| Syntrophaceae_uncultured | 0.00 | 0.00 | 0.00 | 0.00 | 0.00 | 0.00 | 0.00 | 0.00 | 0.00 |
| Syntrophobacteraceae_uncultured | 0.00 | 0.01 | 0.00 | 0.01 | 0.00 | 0.00 | 0.00 | 0.00 | 0.00 |

| | | | | | | | | | |
|---|---|---|---|---|---|---|---|---|---|
| Syntrophomonas | 0.00 | 0.00 | 0.00 | 0.00 | 0.00 | 0.00 | 0.00 | 0.00 | 0.00 |
| Syntrophorhabdus | 0.00 | 0.00 | 0.00 | 0.00 | 0.00 | 0.00 | 0.00 | 0.00 | 0.00 |
| Syntrophus | 0.00 | 0.00 | 0.00 | 0.00 | 0.00 | 0.00 | 0.00 | 0.00 | 0.00 |
| TA18_norank | 0.03 | 0.01 | 0.00 | 0.00 | 0.00 | 0.00 | 0.00 | 0.01 | 0.01 |
| TK34_norank | 0.00 | 0.00 | 0.00 | 0.00 | 0.00 | 0.00 | 0.00 | 0.00 | 0.00 |
| TK85_norank | 0.02 | 0.02 | 0.01 | 0.00 | 0.00 | 0.00 | 0.00 | 0.00 | 0.00 |
| TM6_norank | 0.00 | 0.00 | 0.00 | 0.00 | 0.00 | 0.00 | 0.00 | 0.00 | 0.00 |
| Taibaiella | 0.00 | 0.00 | 0.00 | 0.00 | 0.00 | 0.00 | 0.00 | 0.00 | 0.00 |
| Tenacibaculum | 2.75 | 1.17 | 3.79 | 15.05 | 15.16 | 13.67 | 1.16 | 0.39 | 0.86 |
| Tepidibacter | 0.16 | 0.01 | 0.00 | 0.00 | 0.00 | 0.00 | 0.00 | 0.00 | 0.00 |
| Tepidiphilus | 0.00 | 0.00 | 0.00 | 0.00 | 0.00 | 0.00 | 0.00 | 0.00 | 0.00 |
| Thalassolituus | 0.00 | 0.00 | 0.00 | 0.01 | 0.03 | 0.00 | 0.00 | 0.00 | 0.00 |
| Thalassomonas | 0.00 | 0.02 | 0.00 | 0.03 | 0.04 | 0.08 | 0.00 | 0.00 | 0.00 |
| Thalassospira | 0.00 | 0.00 | 0.00 | 0.00 | 0.00 | 0.00 | 0.00 | 0.00 | 0.03 |
| Thauera | 0.00 | 0.00 | 0.00 | 0.00 | 0.00 | 0.00 | 0.00 | 0.00 | 0.00 |
| Thermonema | 0.00 | 0.00 | 0.00 | 0.00 | 0.00 | 0.00 | 0.00 | 0.00 | 0.00 |
| Thiobacillus | 0.00 | 0.00 | 0.00 | 0.00 | 0.00 | 0.00 | 0.00 | 0.00 | 0.00 |
| Thiohalophilus | 0.00 | 0.00 | 0.00 | 0.00 | 0.00 | 0.00 | 0.00 | 0.00 | 0.00 |
| Thiothrix | 0.13 | 0.08 | 0.05 | 0.02 | 0.00 | 0.01 | 0.05 | 0.03 | 0.03 |
| Thiotrichaceae_unclassified | 0.00 | 0.00 | 0.00 | 0.00 | 0.00 | 0.00 | 0.00 | 0.00 | 0.00 |
| Treponema | 0.00 | 0.00 | 0.00 | 0.00 | 0.00 | 0.00 | 0.00 | 0.00 | 0.00 |
| Truepera | 0.00 | 0.01 | 0.00 | 0.00 | 0.00 | 0.00 | 0.00 | 0.00 | 0.00 |

| | | | | | | | | | |
|---|---|---|---|---|---|---|---|---|---|
| Tumebacillus | 0.01 | 0.01 | 0.01 | 0.01 | 0.01 | 0.01 | 0.01 | 0.00 | 0.00 |
| Turicibacter | 0.00 | 0.00 | 0.00 | 0.00 | 0.00 | 0.00 | 0.00 | 0.00 | 0.00 |
| Ulvibacter | 0.02 | 0.04 | 0.01 | 0.01 | 0.01 | 0.00 | 0.01 | 0.01 | 0.00 |
| Umboniibacter | 0.00 | 0.00 | 0.00 | 0.00 | 0.00 | 0.00 | 0.00 | 0.00 | 0.00 |
| Urania-1B-19_marine_sediment_group | 0.00 | 0.00 | 0.00 | 0.00 | 0.00 | 0.00 | 0.00 | 0.00 | 0.00 |
| Uruburuella | 0.01 | 0.01 | 0.00 | 0.00 | 0.00 | 0.00 | 0.01 | 0.00 | 0.00 |
| VC2.1_Bac22_norank | 0.00 | 0.01 | 0.00 | 0.00 | 0.00 | 0.00 | 0.00 | 0.01 | 0.00 |
| VHS-B3-70_norank | 0.00 | 0.00 | 0.00 | 0.00 | 0.00 | 0.00 | 0.00 | 0.00 | 0.00 |
| Vagococcus | 0.02 | 0.01 | 0.01 | 0.00 | 0.00 | 0.00 | 0.01 | 0.01 | 0.00 |
| Vallitalea | 0.00 | 0.03 | 0.00 | 0.00 | 0.00 | 0.00 | 0.00 | 0.01 | 0.00 |
| Veillonellaceae_uncultured | 0.00 | 0.00 | 0.00 | 0.00 | 0.00 | 0.00 | 0.00 | 0.00 | 0.00 |
| Verrucomicrobiaceae_unclassified | 0.00 | 0.00 | 0.00 | 0.00 | 0.00 | 0.00 | 0.00 | 0.00 | 0.00 |
| Verrucomicrobiaceae_uncultured | 0.00 | 0.01 | 0.00 | 0.00 | 0.00 | 0.00 | 0.00 | 0.00 | 0.00 |
| Vibrio | 19.05 | 18.91 | 30.20 | 10.47 | 10.57 | 19.95 | 36.98 | 45.38 | 35.45 |
| Vibrionaceae_unclassified | 0.00 | 0.01 | 0.00 | 0.00 | 0.00 | 0.00 | 0.00 | 0.00 | 0.00 |
| Vibrionaceae_uncultured | 0.00 | 0.00 | 0.00 | 0.00 | 0.00 | 0.00 | 0.00 | 0.00 | 0.00 |
| Vitellibacter | 0.02 | 0.00 | 0.00 | 0.02 | 0.03 | 0.04 | 0.00 | 0.00 | 0.00 |
| WCHB1-69_norank | 0.00 | 0.00 | 0.00 | 0.00 | 0.00 | 0.00 | 0.00 | 0.00 | 0.00 |
| WD2101_soil_group_norank | 0.00 | 0.00 | 0.00 | 0.00 | 0.00 | 0.00 | 0.00 | 0.00 | 0.00 |
| Weissella | 0.00 | 0.00 | 0.00 | 0.00 | 0.00 | 0.00 | 0.00 | 0.00 | 0.00 |
| Winogradskyella | 0.84 | 0.54 | 0.89 | 2.13 | 1.62 | 2.07 | 0.63 | 0.25 | 0.73 |

| | | | | | | | | | |
|---|---|---|---|---|---|---|---|---|---|
| Xanthomonadaceae_unclassified | 0.00 | 0.00 | 0.00 | 0.00 | 0.00 | 0.00 | 0.00 | 0.00 | 0.00 |
| Xanthomonadaceae_uncultured | 0.00 | 0.00 | 0.00 | 0.00 | 0.00 | 0.00 | 0.00 | 0.00 | 0.00 |
| Xanthomonadales_unclassified | 0.00 | 0.00 | 0.00 | 0.01 | 0.01 | 0.00 | 0.00 | 0.00 | 0.00 |
| Xanthomonadales_uncultured | 0.00 | 0.01 | 0.00 | 0.00 | 0.00 | 0.00 | 0.00 | 0.00 | 0.00 |
| Zeaxanthinibacter | 0.00 | 0.00 | 0.00 | 0.00 | 0.00 | 0.00 | 0.00 | 0.00 | 0.00 |
| Zhangella | 0.00 | 0.00 | 0.00 | 0.00 | 0.00 | 0.00 | 0.00 | 0.00 | 0.00 |
| mitochondria_norank | 0.00 | 0.00 | 0.00 | 0.00 | 0.00 | 0.00 | 0.00 | 0.00 | 0.00 |
| ratAN060301C_norank | 0.00 | 0.00 | 0.01 | 0.00 | 0.01 | 0.00 | 0.00 | 0.00 | 0.00 |
| ss1-B-07-44_norank | 0.00 | 0.00 | 0.00 | 0.00 | 0.00 | 0.00 | 0.00 | 0.00 | 0.00 |
| vadinBA26_norank | 0.00 | 0.00 | 0.00 | 0.00 | 0.00 | 0.00 | 0.00 | 0.00 | 0.00 |
| vadinHA17_norank | 0.00 | 0.00 | 0.00 | 0.00 | 0.00 | 0.00 | 0.00 | 0.00 | 0.00 |
